# Future-Oriented Nanosystems Composed of Polyamidoamine Dendrimer and Biodegradable Polymers as an Anticancer Drug Carrier for Potential Targeted Treatment

**DOI:** 10.3390/pharmaceutics16111482

**Published:** 2024-11-20

**Authors:** Katarzyna Strzelecka, Adam Kasiński, Tadeusz Biela, Anita Bocho-Janiszewska, Anna Laskowska, Łukasz Szeleszczuk, Maciej Gawlak, Marcin Sobczak, Ewa Oledzka

**Affiliations:** 1 Department of Pharmaceutical Chemistry and Biomaterials, Faculty of Pharmacy, Medical University of Warsaw, Banacha 1 Str., 02-097 Warsaw, Poland; katarzyna.strzelecka@wum.edu.pl (K.S.); adam.kasinski@wum.edu.pl (A.K.); marcin.sobczak@wum.edu.pl (M.S.); 2 Department of Polymer Chemistry, Centre of Molecular and Macromolecular Studies, Polish Academy of Sciences, Sienkiewicza 112 Str., 90-363 Lodz, Poland; tadeusz.biela@cbmm.lodz.pl; 3 Faculty of Applied Chemistry, Casimir Pulaski Radom University, Chrobrego 27 Str., 26-600 Radom, Poland; a.janiszewska@uthrad.pl; 4 Department of Pharmaceutical Microbiology, Centre for Preclinical Research, Medical University of Warsaw, Banacha 1b Str., 02-097 Warsaw, Poland; anna.laskowska@wum.edu.pl; 5 Department of Organic and Physical Chemistry, Faculty of Pharmacy, Medical University of Warsaw, Banacha 1 Str., 02-093 Warsaw, Poland; lukasz.szeleszczuk@wum.edu.pl; 6 Laboratory of Physiology and Pathophysiology, Centre for Preclinical Research, Medical University of Warsaw, Banacha 1b Str., 02-097 Warsaw, Poland; maciej.gawlak@wum.edu.pl

**Keywords:** nanopharmacy, camptothecin, PAMAM dendrimer, biodegradable polyesters, controlled drug delivery systems, targeted therapy

## Abstract

**Background/Objectives**: Camptothecin (CPT) is a well-known chemical compound recognized for its significant anticancer properties. However, its clinical application remains limited due to challenges related to CPT’s high hydrophobicity and the instability of its active form. To address these difficulties, our research focused on the development of four novel nanoparticulate systems intended for either oral or intravenous administration. **Methods**: These nanosystems were based on a poly(amidoamine) (PAMAM) dendrimer/CPT complex, which had been coated with biodegradable homo- and copolymers, designed with appropriate physicochemical properties and chain microstructures. **Results**: The resulting nanomaterials, with diameters ranging from 110 to 406 nm and dispersity values between 0.10 and 0.67, exhibited a positive surface charge and were synthesized using biodegradable poly(L-lactide) (PLLA), poly(L-lactide-*co*-*ε*-caprolactone) (PLACL), and poly(glycolide-*co*-*ε*-caprolactone) (PGACL). Biological assessments, including cell viability and hemolysis tests, indicated that all polymers demonstrated less than 5% hemolysis, confirming their hemocompatibility for potential intravenous use. Furthermore, fibroblasts exposed to these matrices showed concentration-dependent viability. The entrapment efficiency (*EE*) of CPT reached up to 27%, with drug loading (*DL*) values as high as 17%. The in vitro drug release studies lasted over 400 h with the use of phosphate buffer solutions at two different pH levels, demonstrating that time-dependent processes allowed for a gradual and controlled release of CPT from the developed nanosystems. The release kinetics of the active compound at pH 7.4 ± 0.05 and 6.5 ± 0.05 followed near-first-order or first-order models, with diffusion and Fickian/non-Fickian transport mechanisms. Importantly, the nanoparticulate systems enabled the stabilization of the pharmacologically active form of CPT, while providing protection against hydrolysis, even in physiological environments. **Conclusions**: In our opinion, these results underscore the promising future of biodegradable nanosystems as effective drug delivery systems (DDSs) for targeted cancer treatment, offering stability and efficacy over short, medium, and long-term applications.

## 1. Introduction

The field of nanotechnology focuses on the molecular creation of a wide range of functional systems. These systems exhibit unique physical, electrical, and optical properties that make them appealing in a wide range of applications [1]. Nanopharmacy is one of the most well-known nanotechnology study areas. It employs nanotechnology to provide precisely tailored pharmaceutical treatments for illness diagnosis, prevention, and therapy. Over the last several decades, there has been an increase in nanopharmacy research, which is now being translated into commercialization efforts around the world, culminating in the selling of a variety of products [2,3]. Drug delivery systems (DDSs) currently dominate nanopharmacy, accounting for more than 75% of total sales [4].

The term DDSs refers to a wide variety of vehicles developed to increase the selectivity of action and improve the drug’s pharmacological and pharmacokinetic characteristics. Most of the drugs in use today are classified as low-molecular-weight compounds. These substances are characterized by their quick metabolism and elimination from the human body, along with a lack of selectivity in their pharmacodynamic effects. This highlights the necessity for the development of formulations that can address these challenges [5]. The idea of DDSs originated as early as 1891, when German scientist Paul Erlich proposed a paradigm for selectively delivering drugs to their site of action, in the right concentration, and at the appropriate time [6].

Traditional ways of drug administration are frequently inefficient and pose a significant risk of side effects. DDSs allow for the alteration of drug characteristics, such as increasing solubility, preventing the drug from being transformed into an inactive form, and improving pharmacokinetic parameters and biodistribution [7]. DDSs are most commonly employed to develop systems that provide controlled or prolonged release of the active substance, known as therapeutic systems. Therapeutic systems are designed to maintain a constant drug concentration throughout time and to make the release rate independent of the amount of drug left in the system (achieving zero-order kinetics). The time required for the therapeutic system to release the drug is highly associated with the type of carrier used, which is the factor that controls the release [2]. The polymeric, inorganic, or composite material that acts as the drug’s carrier must fulfill certain requirements, including biocompatibility, non-toxicity, non-immunogenicity, and no accumulation in the body. Furthermore, its size, surface character, method of binding the active substance, and presence of functional groups on the surface all play an important role in DDSs functioning [8]. 

Recent focus has been directed towards the chemistry of biocompatible and biodegradable polymers, often referred to as “biomedical polymers”. These materials are advantageous due to their ability to be easily hydrolyzed into non-toxic, removable products that can be eliminated through metabolic processes [9,10]. Furthermore, it is essential that biomedical polymers are synthesized with the use of natural initiators, co-initiators, and/or catalysts that are both effective and safe for the environment and human health [11]. Most significantly, these materials have great potential for various medical applications, including tissue engineering, gene therapy, temporary implanted devices, regenerative medicine, nanopharmacy, and coatings on implants [12]. Biodegradable and/or bioresorbable polymers such as polyglycolide (PGA), polylactide (PLA), poly(*ε*-caprolactone) (PCL), poly(trimethylene carbonate) (PTMC) and copolymers of glycolide (GL), L-lactide (LA), *rac*-lactide (*rac*-LA), *ε*-caprolactone (CL), trimethylene carbonate (TMC), or others cyclic esters and carbonates are most commonly used in nanopharmacy to develop DDSs and other therapeutic systems [13]. 

Dendrimers, especially poly(amidoamine) (PAMAM) dendrimers, also known as tree-shaped, stellar, hyperbranched, or cascading polymers, are unique carriers that require particular attention. These are spherical structures with greatly branching arms that terminate in functional groups [14]. The presence of peripheral functional groups (e.g., amine, hydroxyl) as well as internal cavities in PAMAM dendrimers can be adopted to encapsulate drugs or other active compounds. The encapsulation of the active component within the PAMAM dendrimer cavity is a typical idea for drugs with a low molecular weight and poor water solubility. A host-guest interaction occurs inside dendrimer cavities due to the hydrogen bonds between dendrimer and the drug molecules, resulting in a highly soluble complex [15]. Furthermore, the empty spaces in the dendrimer structure are frequently hydrophobic, allowing for interactions with poorly soluble molecules via hydrophobic interactions [16]. The active substance might be additionally conjugated to the peripheral functional groups via covalent bonding. This strategy is preferred, since it provides more precise control over the drug release, enabling its delivery to the target site [17]. Last but not least, the main benefits of using PAMAM dendrimers as drug carriers include improved solubility of hydrophobic drugs, drug protection during transport in the body, a longer period of drug activity, improved pharmacodynamics, reduced side effects, and the possibility of prolonged and controlled drug release [18].

Cancer is a prevalent cause of mortality worldwide. The American Cancer Society predicts that in 2024, 2,001,140 new cancer cases and 611,720 cancer deaths will occur in the United States [19]. According to the National Cancer Registry data, the overall number of malignant tumor cases in Poland in 2020 was over 146,000, with about 100,000 deaths. Unfortunately, the disease structure in Poland is remarkably similar to that of other countries [20]. 

The main objective of oncological therapy is to bring the tumor under control to ensure better life expectancy and quality. In addition to surgical therapy and radiotherapy, chemotherapy includes a wide range of drugs from various groups, each with a different mechanism of action. All three treatment procedures carry a significant risk of major side effects, as well as limited specificity and selectivity [21]. 

Chemotherapeutic drug treatment is associated with high toxicity, which is a severe clinical issue that has a considerable impact on the patient’s quality of life [22]. Furthermore, the anticancer drugs utilized exhibit rapid renal clearance, making it difficult to achieve the appropriate therapeutic concentration, thus necessitating the use of higher doses. Also, the majority of drugs taken systemically are lipophilic, tend to accumulate in the liver, and, as a result, may lead to hepatic steatosis [23]. In order to improve the efficacy and safety of therapy, scientists are searching for carriers that would allow targeted drug delivery to cancer cells as well as controlled drug release.

One of the most promising anticancer drugs is camptothecin (CPT), a pentacyclic alkaloid isolated from the *Camptotheca acuminata* tree [24]. The α-hydroxylactone ring in the CPT’s structure plays a crucial role in sustaining the anticancer efficiency, since its opening leads to deactivation [25]. CPT provides a high cytotoxic effect, and its mechanism of action relies on the suppression of topoisomerase I activity, which results in DNA damage and, consequently, cell death [26]. Nonetheless, CPT’s therapeutic usage, however, is challenging due to its low solubility in water, high cellular toxicity, and hydrolytic cleavage of the lactone ring to the inactive carboxyl form in vivo [27]. In recent years, extensive research has been conducted to generate novel formulations of CPT, which could address the aforementioned concerns [24]. It involves the development of polymer nanoparticles, micelles, liposomes, and lipid nanoparticles [28,29,30,31,32]. Another alternative strategy is to encapsulate CPT inside the cavity of a PAMAM (polyamidoamine) dendrimer, which has minimal cytotoxicity and is able to penetrate biological membranes [33]. 

In our previous investigation, we examined PAMAM dendrimer/CPT complex for non-small-cell lung cancer cell targeting [34]. Considering that the findings we obtained were highly promising and revealed an opportunity for further study, we decided to develop innovative PAMAM dendrimer/biodegradable polymer nanosystems as a CPT carrier in the present research. To date, there have been no reports in the literature of nanosystems that encapsulate both CPT and its derivatives inside a PAMAM dendrimer, with a biodegradable polymer serving as the carrier. We suspected that encapsulating CPT inside a PAMAM dendrimer would improve its solubility and the therapeutic bioavailability, while utilizing a biodegradable material with a predicted microstructure and topology would allow for controlled and sustained drug release. Furthermore, the acidic microenvironment formed by the hydrolytic degradation of the biodegradable matrix will inhibit the active lactone form of CPT from converting to its inactive carboxyl form. As a result, the developed novel nanosystems may allow for oral or intravenous administration, while maintaining the therapy’s effectiveness and biosafety.

## 2. Materials and Methods

### 2.1. Materials

(*S*)-(+)-Camptothecin ((*S*)-4-Ethyl-4-hydroxy-1H-pyrano-[3′,4′:6,7]indolizino [1,2-b]quinoline-3,14(4H,12H)-dione) (CPT) (purity: >97.0% (HPLC)) was purchased from TCI Europe N.V. (Zwijndrecht, Belgium). PAMAM dendrimer, ethylenediamine core, generation 4.0, 10 wt.% solution in methanol was obtained from Merck, Poznan, Poland. L-lactide ((3S)-*cis*-3,6-dimethyl-1,4-dioxane-2,5-dione) (LA) (purity: 100%), ε-caprolactone (2-oxepanone) (CL) (purity: 100%), glycolide (1,4-dioxane-2,5-dione) (GL) (purity: 100%), diethylzinc solution 1.0 M in hexanes (Et_2_Zn), poly(ethylene glycol) average *M*_n_ 400 (PEG-400), Tween^®^ 80 (polyethylene glycol sorbitan monooleate, polysorbate 80) were also obtained from Merck, Poznan, Poland. *N*,*N*-dimethylformamide (DMF, anhydrous, 99%, Avantor Performance Materials S.A., Gliwice, Poland), dimethyl sulphoxide (DMSO, anhydrous, 99%, Avantor Performance Materials S.A., Gliwice, Poland), methanol (anhydrous, 99.9%, Avantor Performance Materials S.A., Gliwice, Poland), dichloromethane (DCM, anhydrous, 99.8%, Chempur, Piekary Śląskie, Poland), chloroform (pure, stabilized with amylen, 98.5%, Chempur, Piekary Śląskie, Poland), petroleum ether boiling range 60–90 °C (Chempur, Piekary Śląskie, Poland), liquid paraffin (Chempur, Piekary Śląskie, Poland), hydrochloric acid (≥37%, Merck, Poznan, Poland) and acetonitrile ((ACN), anhydrous, 99.8%, Avantor Performance Materials S.A., Gliwice, Poland) were used as received. Dimethyl-*d*_6_-sulfoxide (DMSO-*d*_6_) in ampoules, for NMR measurements (99.9 atom% D), was purchased from ARMAR Chemicals (Döttingen, Switzerland), whereas chloroform-d (deuterochloroform, CDCl_3_ 99.8 atom% D) was purchased from Merck, Poznan, Poland. Phosphate buffer solutions (PBS, pH 7.40 ± 0.05 and pH 6.50 ± 0.05, 20 °C, Avantor Performance Materials S.A., Gliwice, Poland) were also used as received.

### 2.2. Synthesis of PAMAM Dendrimer/CPT Complex

The synthesis was carried out according to a previously established method by Oledzka et al. [34]. Briefly, 9.1 mg of CPT was placed in a 5.0 mL round-bottom flask and dissolved in 2 mL of DMF. Then, 1 mL of PAMAM dendrimer (G4, 10 wt.% in methanol) was added, and the prepared reaction mixture was incubated in the dark for 24 h at 37 °C, at a rotational speed of 130 rpm. Following that, the DMF was evaporated, and 2.0 mL of distilled water was added to the solid residue and stirred for 1 h in order to remove the unreacted CPT. The obtained suspension was filtered using a 0.45 μm syringe filter, and the supernatant was evaporated using a rotary evaporator. The resulting solid compound was dried in a vacuum oven at 40 °C for 72 h, until the constant weight was achieved. The PAMAM dendrimer/CPT complex synthesis yielded 84.85%. The synthesised complex was stored in the dark in an inert gas atmosphere until it was used. Subsequent analyses were conducted within 24 h of synthesis.

The ^1^H NMR spectrum of the synthesized PAMAM dendrimer/CPT complex (DMSO-*d*_6_, 400 MHz; δH): 8.69 ppm (G, B-ring of CPT), 7.93 ppm (H and K, A-ring of CPT), 7.70 ppm (I and J, A-ring of CPT), 7.34 ppm (A, D-ring of CPT), 5.42 ppm (E, E-ring of CPT), 5.29 ppm (F, C-ring of CPT), 4.87 ppm (6 (-CH_2_-CH_2_-C(O)-N(H)-) of PAMAM dendrimer), 4.67 ppm (7 (-C(O)-N(H)-CH_2_-CH_2_-NH_2_) of PAMAM dendrimer), 2.00–3.62 ppm (2 (-CH_2_-CH_2_-C(O)-N(H)-), 3 (-CH_2_-CH_2_-C(O)-N(H)-), 4 (-C(O)-N(H)-CH_2_-CH_2_-) and 5 (-C(O)-N(H)-CH_2_-CH_2_-NH_2_) of PAMAM dendrimer), 1.86 ppm (B, E-ring of CPT, methylene group), 1.22 ppm (1 (-N-CH_2_-CH_2_-N-) and 1′ (-C(O)-N(H)-CH_2_-CH_2_-) of PAMAM dendrimer), 0.87 ppm (C, E-ring of CPT, methylene group) (Figure S1, Supplementary Materials).

### 2.3. Synthesis of Biodegradable Polymers

The Ring-Opening Polymerisation (ROP) process was applied to produce four polymers, with ZnEt_2_ acting as a catalyst and PEG-400 serving as a co-initiator. The reactions were carried out in bulk. Briefly, carefully weighted amounts of monomers—LA (M1), LA and CL (M2), and CL and GL (M3, M4) (1 g total)—were placed in glass ampoules and vacuum dried to remove trace amounts of moisture. After that, calculated amounts of ZnEt_2_ and PEG-400 were added in an argon atmosphere via the Schlenk line, and the ampoules were safely sealed. Reactions were carried out in an oil bath at 130 °C for 24 h. The post-reaction mixtures of M1 and M2 were then dissolved in 6.0 mL of DCM, while M3 and M4 were dissolved in 6.0 mL of a 1:1 mixture consisting of DCM and chloroform. Following that, the dissolved polymers were transferred to beakers and precipitated using an ice-cold 5% HCl solution in methanol. The precipitates were subsequently washed twice more with pure, ice-cold methanol. The collected products were vacuum-dried for 24 h at 30 °C.

The ^1^H NMR spectrum of the synthesized PLLA (M1, CDCl_3_, 400 MHz; δH): 5.15 ppm (2, (-O(O)C-(H)C(CH_3_)-)), 4.31 ppm (5, (-O(O)C-(H)C(CH_3_)-OH)), 3.63 ppm (PEG, (-CH_2_-CH_2_-)), 2.17 ppm (1, (-O(O)C-(H)C(CH_3_)-OH)), 1.42 ppm (3, (-O(O)C-(H)C(CH_3_)-)), 1.25 ppm (4, (-O(O)C-(H)C(CH_3_)-OH)) (Figure S2, Supplementary Materials).

The ^13^C NMR spectrum of the synthesized PLLA (M1, CDCl_3_, 400 MHz; δH): 169.7 ppm (-O(O)C-(H)C(CH_3_)-), 69.0 ppm (-O(O)C-(H)C(CH_3_)-), 16.7 ppm (2, (-O(O)C-(H)C(CH_3_)-)) (Figure S3, Supplementary Materials).

The ^1^H NMR spectrum of the synthesized PLACL (M2, CDCl_3_, 400 MHz; δH): 5.15 ppm (2, (-O(O)C-(H)C(CH_3_)-)), 5.06 ppm (3, (-O-(H)C(CH_3_)-C(O)O-(CH_2_)_5_-)), 4.12 ppm (5, (-(H)C(CH_3_)-C(O)O-CH_2_-CH_2_-CH_2_-CH_2_-CH_2_-C(O)O-)), 4.05 ppm (10, (-(CH_2_)_5_-C(O)O-CH_2_-CH_2_-CH_2_-CH_2_-CH_2_-)), 3.64 ppm (PEG, (-CH_2_-CH_2_-)), 2.38 ppm (9, (-(H)C(CH_3_)-C(O)O-CH_2_-CH_2_-CH_2_-CH_2_-CH_2_-C(O)O-)), 2.29 ppm (14, (-(CH_2_)_5_-C(O)O-CH_2_-CH_2_-CH_2_-CH_2_-CH_2_-)), 1.45–1.71 ppm (1, (-O(O)C-(H)C(CH_3_)-), 4, (-O-(H)C(CH_3_)-C(O)O-(CH_2_)_5_-), 6, (-(H)C(CH_3_)-C(O)O-CH_2_-CH_2_-CH_2_-CH_2_-CH_2_-C(O)O-), 8, (-(H)C(CH_3_)-C(O)O-CH_2_-CH_2_-CH_2_-CH_2_-CH_2_-C(O)O-), 11, (-(CH_2_)_5_-C(O)O-CH_2_-CH_2_-CH_2_-CH_2_-CH_2_-), 13 (-(CH_2_)_5_-C(O)O-CH_2_-CH_2_-CH_2_-CH_2_-CH_2_-)), 1.39 ppm (7, (-(H)C(CH_3_)-C(O)O-CH_2_-CH_2_-CH_2_-CH_2_-CH_2_-C(O)O-), 12, (-(CH_2_)_5_-C(O)O-CH_2_-CH_2_-CH_2_-CH_2_-CH_2_-)) (Figure S4, Supplementary Materials).

The ^13^C NMR spectrum of the synthesized PLACL (M2, CDCl_3_, 400 MHz; δH): 172.6–173.9 ppm (1, (-(CH_2_)_5_-C(O)O-(CH_2_)_5_-), carbonyl carbon atoms of oxycaproyl unit), 169.3–171.2 ppm (2, (-O(O)C-(H)C(CH_3_)-), carbonyl carbon atoms of lactidyl unit), 67.8–69.9 ppm (4, (-O(O)C-(H)C(CH_3_)-), methine carbon atoms of lactidyl unit), 63.4–65.9 ppm (9, (-C(O)O-CH_2_-CH_2_-CH_2_-CH_2_-CH_2_-), ε-carbon atoms of oxycaproyl unit), 33.1–33.4 ppm (5, (-C(O)O-CH_2_-CH_2_-CH_2_-CH_2_-CH_2_-), α-carbon atoms of oxycaproyl unit)), 28.2 ppm (8, (-C(O)O-CH_2_-CH_2_-CH_2_-CH_2_-CH_2_-), δ-carbon atoms of oxycaproyl unit), 23.9–25.9 ppm (6, (-C(O)O-CH_2_-CH_2_-CH_2_-CH_2_-CH_2_-), β-carbon atoms of oxycaproyl unit, 7, (-C(O)O-CH_2_-CH_2_-CH_2_-CH_2_-CH_2_-), γ-carbon atoms of oxycaproyl unit), 16.6 ppm (3, (-O(O)C-(H)C(CH_3_)-), methyl carbon atoms of lactidyl unit) (Figure S5, Supplementary Materials).

The ^1^H NMR spectrum of the synthesized PGACL (M3 and M4, CDCl_3_, 400 MHz; δH): 4.60 ppm (1, (-O-CH_2_-C(O)-), glycolidyl unit), 4.05 ppm (2, (-C(O)O-CH_2_-CH_2_-CH_2_-CH_2_-CH_2_-), caproyl unit), 3.65 ppm (PEG, (-CH_2_-CH_2_-)), 2.30 ppm (6, (-C(O)O-CH_2_-CH_2_-CH_2_-CH_2_-CH_2_-), caproyl unit), 1.65 ppm (6, (-C(O)O-CH_2_-CH_2_-CH_2_-CH_2_-CH_2_-), caproyl unit, 5, (6, (-C(O)O-CH_2_-CH_2_-CH_2_-CH_2_-CH_2_-), caproyl unit), 1.38 ppm (4, (-C(O)O-CH_2_-CH_2_-CH_2_-CH_2_-CH_2_-), caproyl unit) (Figure S6, Supplementary Materials).

The ^13^C NMR spectrum of the synthesized PGACL (M3 and M4, CDCl_3_, 400 MHz; δH): 172.8–173.5 ppm ((-O-CH_2_-C(O)-), glycolidyl unit), 167.9 ppm ((-C(O)O-CH_2_-CH_2_-CH_2_-CH_2_-CH_2_-), caproyl unit), 64.2–65.2 ppm ((-C(O)O-CH_2_-CH_2_-CH_2_-CH_2_-CH_2_-), ε-carbon atoms of oxycaproyl unit), 60.2–60.5 ppm ((-O-CH_2_-C(O)-), glycolidyl unit), 33.6–34.1 ppm ((-C(O)O-CH_2_-CH_2_-CH_2_-CH_2_-CH_2_-), α-carbon atoms of oxycaproyl unit)), 28.2 ppm ((-C(O)O-CH_2_-CH_2_-CH_2_-CH_2_-CH_2_-), δ-carbon atoms of oxycaproyl unit), 25.4 ppm ((-C(O)O-CH_2_-CH_2_-CH_2_-CH_2_-CH_2_-), γ-carbon atoms of oxycaproyl unit), 24.4 ppm ((-C(O)O-CH_2_-CH_2_-CH_2_-CH_2_-CH_2_-), β-carbon atoms of oxycaproyl unit) (Figure S7, Supplementary Materials).

The conversion of monomers was determined from ^1^H NMR spectra of post-reaction, by comparing the integrated signals of equivalent protons from the monomer and polymer, according to the following formula (Equation (1)):

(1) $${Conv}_{i}=\frac{I_{i}}{I_{i}+I_{I}}$$

where *I_i_* and *I_I_* represent the integral intensities of signals from equivalent protons in the monomer and polymer, respectively.

The microstructural analysis of the obtained polymers was carried out with ^13^C NMR spectra in the methine and carbonyl regions for PLLA and the carbonyl region for PLACL. The microstructure of PGACL matrices was examined using ^1^H NMR spectra in the region of methylene protons of glycolidyl units (*GG*) and ε-methylene proton region of caproyl units (*Cap*). By comparison with the available literature, relevant spectral lines were assigned to the respective sequences [35,36,37].

With the use of ^1^H and ^13^C spectra, the experimental lengths of glycolidyl units (*L*^e^_GG_) and lactydyl units (*L*^e^_LL_) were calculated according to Equation (2), whereas experimental lengths of caproyl units were calculated with the use of Equation (3) for ^1^H NMR spectrum and Equation (4) for ^13^C NMR spectrum, respectively:

(2) $$L_{XX}^{e}=\frac{CapXCap+CapXX+XXCap+XXX}{CapXCap+\frac{1}{2}(CapXX+XXCap)}\times \frac{1}{2}$$

(3) $$L_{Cap}^{e}=\frac{XCap+CapCap}{XCap}$$

(4) $$L_{Cap}^{e}=\frac{CapCapCap+XCapCap+CapCapX+XCapX}{XCapX+\frac{1}{2}(XCapCap+CapCapX)}$$

where X represents lactydyl unit -OCH(CH_3_)CO- (*L*) or glycolidyl unit -OCH_2_CO- (*G*), *Cap* represents caproyl unit -O(CH_2_)_5_CO-, and *CapXX*, *CapXCap*, *XCap*, *CapCapX*, and so on represent particular sequences in the polymeric chain.

The transesterification of the second mode (*T*_II_) may cause a break in the *L* or *G* units within the copolymeric chain, resulting in the formation of distinct *CapLCap* or *CapGCap* sequences. To quantitatively determine the yield of *T*_II_ in the copolymeric chains, the following Equation (5) was applied:

(5) $$T_{II}=\frac{\left[CapXCap\right]}{{\left[CapXCap\right]}_{R}}$$

where [*CapXCap*] represents the experimental concentration of *CapXCap* sequences, and [*CapXCap*]_R_ is the concentration of *CapXCap* sequences in a completely random copolymeric chain.

The [*CapXCap*]_R_ can be described using the following formula (Equation (6)), where the ratio of [Cap]/[X] is denoted as k′:

(6) $${\left[CapXCap\right]}_{R}=\frac{k^{\prime 2}}{{{(k}^{\prime }+1)}^{3}}$$

The degree of the randomness (*R*) of the copolymeric chain was calculated according to Equation (7):

(7) $$R=\frac{L_{XX}^{R}}{L_{XX}^{e}}$$

where L^R^_XX_ represents the average lengths of lactydyl or glycolidyl blocks (Equation (8)) and caproyl blocks (Equation (9)), respectively, in a completely randomized copolymeric chain.

(8) $$L_{XX}^{R}=\frac{k^{\prime }+1}{{2k}^{\prime }}$$

(9) $$L_{Cap}^{R}=k^{\prime }+1$$

### 2.4. In Vitro Hydrolytic Degradation of Polymerics

In brief, 10 mg of the produced polymers were weighed into prepared and labelled glass vials. The experiment was conducted simultaneously in two series: 10.0 mL of buffer pH 7.4 ± 0.05 was added to the first series, and 10.0 mL of buffer pH 6.5 ± 0.05 was used in the second series. The prepared samples were placed in an incubator set to 37 °C with a rotational speed of 100 rpm. The measurement points for each sample were set at 7, 14, 21, 28, 56, and 77 days. The pH of the sample was determined at each time point using the Volcraft pH-100ATC pH-meter.

### 2.5. Synthesis of the Nanosystems Composed of PAMAM Dendrimer/CPT Complex and Biodegradable Polymer

The beaker was filled with 49.5 mL of liquid paraffin and 0.5 mL of Tween 80 to produce a 0.5% solution, which was then placed on a magnetic stirrer. Two glass vials were weighed with 200 mg of the proper biodegradable polymer (M1, M2, M3, or M4) and 20 mg of the PAMAM/CPT complex. The appropriate amount of DCM was poured to the vials with the matrices, followed by methanol to the vial with complex. All vials spent 30 min in an ultrasonic cleaner. The contents of the vials were then mixed and withdrawn using a syringe. The contents of the syringe were added in a dropwise manner to the Tween 80 solution in liquid paraffin at a constant mixing speed of 1000 rpm. Subsequently, the beaker was placed in an ultrasonic homogeniser for 5 min, while cooling in an ice bath. The beaker was then put back on the magnetic stirrer at 400 rpm, covered with parafilm, and left for about 3 h. Following this, n-hexane was poured in the beaker and kept at room temperature for 24 h. The precipitate solution was then decanted, and the resulting products (denoted as NP1, NP2, NP3, NP4) were washed seven times with petroleum ether before drying.

To determine the drug loading (*DL*) and the entrapment efficiency (*EE*) of CPT in the obtained nanosystems, the accurately weighted amounts of the developed products (~10 mg) were dissolved in 5 mL of ACN and sonicated for 30 min in an ice bath. The resulting solutions were filtered through 0.45 µm syringe filters to the HPLC vials.

The *DL* was calculated using the following Equation (10):

(10) $$DL=\frac{Measured\;CPT\;content}{Nanosystem\;weight}\times 100\%$$

whereas the *EE* was determined using Equation (11):

(11) $$EE=\frac{Measured\;CPT\;content}{Theoretical\;CPT\;content}\times 100\%$$

### 2.6. In Vitro Release Study of CPT from the Developed Nanosystems

The obtained nanosystems (~50 mg) were suspended in PBS at pH 6.50 ± 0.05 and 7.40 ± 0.05. The resulting suspensions were transferred to dialysis membranes with pore diameters of 3.50 kDa and immersed in appropriate buffer solution. The release experiment lasted 23 days and involved constant rotating at 100 cycles per min at 37 °C. At each time point, 1.5 mL of samples were collected and supplemented with 1.5 mL of fresh pH-appropriate PBS. The collected samples were then subjected to HPLC analysis to assess the amount of CPT released as well as the percentage amount of lactone and carboxylic form of CPT. The analysis was performed using gradient elution and UV detector at a wavelength of 363 nm. The calibration curve was designed between 0.46 and 46.55 μg/mL (R^2^ = 1.000, y = 2.3926x − 0.2040). Each of the collected samples was tested three times, and the outcome of the analysis was expressed as an amount of CPT released (in μg).

### 2.7. Mathematical Models

The release data points were subjected to zero-order, first-order kinetics, Higuchi, and Korsmeyer–Peppas models, respectively. The calculations were performed according to the formulas provided below:

Zero-order:
  (12) F=kt
First-order:
  (13) $$\log F=\log F_{0}-\frac{kt}{2.303}$$
Higuchi model:
  (14) $$F=k\sqrt{t}$$
Korsmeyer–Peppas model:
  (15) $$F=kt^{n}\;(F<0.6)$$
  where:
*F* is the fraction of drug released from the matrix after time *t*;
*F*_0_ is the initial amount of the drug;
*k* is a model constant; and *n* is drug release exponent in the Korsmeyer–Peppas model [34,38,39,40,41].

### 2.8. Hemolysis

Defibrinated sheep blood was purchased from Graso, Starogard Gdański, Poland. Blood was centrifuged at 2000 rpm for 10 min (RT). The supernatant was carefully removed without disturbing the RBC pellet. Samples were resuspended in 5 mL of PBS (pH 7.40 ± 0.05) and centrifuged at 2000 rpm for 10 min. The washing step was repeated three times until the supernatant was clear. The remaining RBC pellet was diluted in PBS (pH 7.4, RT) to obtain a 2% suspension. Next, 2% RBC suspension was incubated with serial dilution of the proper matrix (M1, M2, M3, or M4, 0.05–0.5 mg/mL) in a 1:1 ratio for 1 h at 37 °C. Positive control (100% hemolysis) was prepared with distilled water and negative control (0% hemolysis) with PBS (pH 7.40 ± 0.05). Next, samples were centrifuged at 2000 rpm for 5 min (RT), and 100 µL of supernatant from each sample was transferred to a 96-well F-bottom plate. Optical density (OD) was measured at 540 nm in a microplate reader (BioTek Synergy HTX Multimode Reader, Agilent, Santa Clara, CA, USA). The value of compound-induced hemolysis was calculated according to Equation (16) [42]:

Hemolysis [%] = (A − A_0%_)/(A_100%_ − A_0%_)·100% (16)

where A is the absorbance of the sample, A_100%_ is the absorbance of the positive control (100% hemolysis), and A_0%_ is the absorbance of the negative control (0% hemolysis).

### 2.9. Cell Culture and Viability Assay

Normal human dermal fibroblasts (Promocell) were maintained in Dulbecco’s Modified Eagle Medium (DMEM) (Biowest, Nuaillé, France) containing 10% fetal bovine serum (FBS) (Biowest, Nuaillé, France) and 1% penicillin-streptomycin solution (Biowest, Nuaillé, France). Cells were incubated at 37 °C in humidifying conditions with 5% CO_2_. At confluency of 80% cells were harvested and seeded in 96-well plates at a density of 3·10^3^ per well and incubated overnight. The growth media was then changed to DMEM at pH 7.40 ± 0.05 or DMEM at pH 6.50 ± 0.05 with serial dilutions of M1, M2, M3, or M4 (0.05–50 µg/mL). Plates were incubated at 37 °C for 72 h. After the incubation period, 20 µL of CellTiter 96^®^ AQueous One Solution Cell Proliferation Assay (MTS) (Promega, Walldorf, Germany) was added to each well, and the plates were incubated for an additional 2 h at 37 °C. The absorbance was measured at ƛ = 490 nm. The viability of untreated cells (negative control) was defined as 100%.

### 2.10. Statistical Analysis

The in vitro experiment findings were provided as a mean and standard deviation of mean (SEM). The normality of distribution was tested first, followed by one-way ANOVA or the non-parametric Kruskal–Walli’s test to determine the statistical significance of mean differences. To compare the different circumstances, two-way ANOVA was employed, followed by Bonferroni’s multiple comparisons. *p* < 0.05 was deemed statistically significant. The data was analysed with GraphPad Prism 5.0 (GraphPad Software, San Diego, CA, USA).

### 2.11. Molecular Modelling of the Nanosystems Composed of PAMAM Dendrimer/CPT Complex and Biodegradable Polymer

The structure of PAMAM dendrimer generation 4.0 was built using the Build Polymers module within the Biovia Materials Studio 2020 software suite (https://www.3ds.com/products-services/biovia/products/molecular-modeling-simulation/biovia-materials-studio/, accessed on 20 October 2024). The terminal, primary amino groups of dendrimers are demonstrated to be protonated and thereby positively charged at pH ranges between 6.5 ± 0.05 and 9.0 ± 0.05 [43]. Since the dissolution analyses conducted for this study were done at pH 6.5 ± 0.05 and 7.4 ± 0.05, all of the terminal amino groups of the modeled PAMAM structure have been protonated to improve comparability with those studies.

The Forcite Plus module has been used to optimize the structures, applying ultra-fine-quality settings of geometry optimization and smart algorithm. The convergence tolerance values were set to 2 · 10^−5^ kcal/mol for energy, 1 · 10^−3^ kcal/(Å · mol) for force, and 1 · 10^−5^ Å for displacement, with 5 · 10^4^ maximum iterations. All calculations have been performed using the COMPASS II forcefield [44], which has been shown to provide accurate results when modeling PAMAM dendrimers [45]. As charge assignments are one of the characteristics provided in this forcefield specification, the charges have been allocated using the COMPASS II forcefield. The electrostatic and van der Waals summation methods were both atoms-based with cubic spline truncation of non-bond energy terms, 18.5 Å cutoff distance, 1 Å spline width, and 0.5 Å buffer width.

Molecular docking calculations were conducted utilizing the Adsorption Locator module, a part of the Biovia Materials Studio 2020 software suite. The Adsorption Locator module identifies potential adsorption sites through Monte Carlo searches of the configu-rational space of the substrate (PAMAM) and adsorbate (CPT) system, as the temperature is gradually lowered during the simulated annealing process of a molecular dynamics simulation. This approach offers the advantage of allowing for the simultaneous docking of the designated quantities of CPT, specifically three molecules of API in this study. The Adsorption Locator makes use of a simulation of annealing with geometry adjustments in-between repeating heat-cool cycles. For repeatable results, 10 cycles with 100,000 steps each and annealing temperatures ranging from 100 to 5000 K were utilized. For “conformer”, “rotate”, and “translate”, the Monte Carlo parameters were set to a probability of 0.32 (ratio = 1), whereas “regrow” was set to 0.1 (ratio = 0.03). The computations employed the identical geometry optimization parameters from the preceding paragraph’s detailed description of geometry optimization and the COMPASS II force field.

To calculate the stoichiometry of the PAMAM dendrimer/CPT complex, the formula below was used (Equation (17)):

(17) $$L=\frac{m_{CPT}\times M_{PAMAM}\times EE}{M_{CPT}\times V_{PAMAM}\times d_{PAMAM}\times C_{PAMAM}}=3.1$$

where L represents the loading (average number of CPT molecules per one PAMAM molecule), m_CPT_ denotes the mass of added CPT (9.1 · 10^−3^ g), M_PAMAM_ indicates the molar mass of PAMAM (14,214.17 g/mol), *EE* refers to the encapsulation efficiency (84.85%), M_CPT_ signifies the molar mass of CPT (348.35 g/mol), V_PAMAM_ is the volume of added PAMAM methanol solution (1 · 10^−3^ dm^3^), d_PAMAM_ represents the density of the PAMAM methanol solution (0.813 g/cm^3^), and C_PAMAM_ is the percentage concentration of PAMAM methanol solution (10%).

The optimised systems, which included three molecules of CPT, were then analysed using molecular dynamics (MD) simulations with periodic boundary conditions [34]. First, the MODELS 1–4 were created using the Amorphous Cell module of Materials Studio. This application provides a comprehensive set of tools to construct three-dimensional periodic structures of polymeric systems. The module builds molecules in a cell in a Monte Carlo fashion, by minimizing close contacts between atoms, whilst ensuring a realistic distribution of torsion angles for any given forcefield. The output of such simulations is a single periodic structure, which in the case of the present study served as the basic input to MD simulations. The composition of the cubic unit cells of MODELS 1–4 included PAMAM G4.0/3 molecules of CPT complex, surrounded by the polymer. After construction, the systems MODELS 1–4 were subjected to geometry optimization, including unit cell dimensions optimization, using the parameters from the preceding paragraph’s detailed description of geometry optimization setup and the COMPASS II force field as well.

A simulated annealing process was conducted in five cycles of 10,000 steps, within a temperature range of 300–500 K, utilizing the NVT ensemble mode, five heating ramps per cycle, 100 dynamics steps per run, and the Berendsen thermostat with a 0.1 ps decay constant. A production run lasting 100 ns was conducted in NVT mode, utilizing the Nosé–Hoover thermostat for temperature regulation, with a Q-ratio set at 2 and a time step of 1 fs. The MD calculations employed the COMPASS II force field, utilizing parameters identical to those applied during geometry optimization.

### 2.12. Measurements

All ^1^H and ^13^C NMR measurements were performed using a Varian 300 MHz (Palo Alto, Santa Clara, CA, USA) and Agilent Technologies 400 MHz (Santa Clara, CA, USA) spectrometer.

The quantitative analysis of CPT content in the samples was determined using previously developed and described method by Oledzka et al. [34]. The study was performed using HPLC apparatus (Beckman Coulter, Miami, Florida, USA) equipped with a UV/Vis detector (Beckman Coulter System Gold^®^ 166, Fullerton, CA, USA), an autosampler (Triathlon 900, Spark Holland B.V., Emmen, Netherlands) and a pump (Beckman Coulter System Gold^®^ 125NM Solvent Module, Fullerton, CA, USA). The C18 column (Luna 25 cm, 5 μm, 100 A, Phenomenex, Basel, Switzerland) was placed at 30 °C, and the injection volume was 20 μL. The gradient mobile phase consisting of acetonitrile (ACN): phosphate buffer solution (pH 6.50 ± 0.05 or 7.40 ± 0.05) (*v*/*v*) was delivered at a flow rate of 1.0 mL/min (ACN concentration varied with time: 5% ACN after 0 min, 15% ACN after 5 min, 35% ACN after 15 min, 50% ACN after 20 min, and 5% ACN after 22 and 25 min). The retention time of CPT’s carboxylic form was 13.90 ± 0.1 min and 20.69 ± 0.1 min for the lactone form of CPT.

The number-average molecular weight (*M*_n_) and dispersity index (*Ð*) of the polymeric materials were measured by the SEC-MALLS instrument (Wyatt Technology Corporation, Santa Barbara, CA, USA), composed of an autosampler, degasser, 1100 Agilent isocratic pump, thermostatic box for columns, a photometer MALLS DAWN EOS (Wyatt Technology Corporation, Santa Barbara, CA, USA), and differential refractometer Optilab Rex (Wyatt Technology Corporation, Santa Barbara, CA, USA). For data collecting and processing, the ASTRA 4.90.07 software (Wyatt Technology Corporation, Santa Barbara, CA, USA) was used. For separation the two 2 × PLGel 5 microns MIXED-C columns were used. The samples were injected as a solution in methylene chloride, and the volume of the injection loop was 100 mL. The mobile phase consisting of methylene chloride was delivered at a flow rate of 0.8 mL/min.

The dynamic light scattering studies of hydrodynamic diameters and zeta potential were performed using the Zetasizer Nano ZS (Malvern, UK). The excitement wavelength of the DLS instrument was 633 nm (He-Ne laser, power = 5 W), and the measurement angle was 173. Polystyrene disposable cuvettes were used during hydrodynamic diameter determination, while the zeta potential measurements were performed using standard dip cells equipped with palladium electrodes. The assessments were conducted at 25 °C in aqueous solutions, using an excitation wavelength of 633 nm (He-Ne laser, power = 5 W) of DLS instrument. Prior to measurement, the samples were briefly sonicated. All experiments were replicated three times.

Transmission electron microscopy (TEM) was used to assess the shape morphology and size of the developed nanosystems. The developed products that had been synthesized were collected on TEM grids. TEM studies were carried out using equipment installed in the Laborarory of Electron Microscopy, Nencki Institute of Experimental Biology, Polish Academy of Sciences, Warsaw, Poland. Electron micrographs were taken using a CCD MORADA G2 (EMSIS GmBH, Münster, Germany) camera on a JEM 1400 (JEOL Co., Tokyo, Japan) transmission electron microscope sponsored by the EU Structural Funds: Centre of Advanced Technology BIM—Equipment purchase for the Laboratory of Biological and Medical Imaging.

Concerning confocal fluorescence microscopy, the samples were mounted (Chemland, Stargard, Poland) and sealed with #1.5 coverslips (Epredia, Braunschweig, Germany). The fluorescent signal was acquired with an Olympus Fluoview FV1000 confocal microscope (EVIDENT Europe GmbH, Hamburg, Germany) and with 10× and 60× objectives. Fluorescence was excited with 488 nm line of an Argon laser, and the emission was detected at 461 nm.

## 3. Results and Discussion

### 3.1. Synthesis and Characterization of Biodegradable Polymers

As a first approach, four biodegradable homo- and copolymers were synthesized via ROP of LA, CL, and GL in the presence of ZnEt_2_ as a catalyst and PEG-400 as a biosafe co-initiator. The polymerization process was carried out at 130 °C for 24 h. The molar ratio of the monomers to the catalyst and co-initiator was constant and equal to 100:1:1. The reactions were carried out in bulk. The polymers were synthesized with a moderate high monomer conversion, satisfactory yield, reasonably narrow *Ɖ*, and *M*_n_ ranged from 8700 to 21,300 g/mol (Table 1). It is well established in the scientific literature that polymeric matrices characterized by a higher *M*_n_ tend to demonstrate slower drug release profiles compared to those with lower *M*_n_ values [46]. Therefore, our primary intention in this study was to develop PAMAM dendrimer/biodegradable polymer nanosystems that are proficient in releasing CPT in a controlled manner over varying durations, including short, medium, and long periods.

The ^1^H and ^13^C NMR spectra of the synthesized materials were utilized to determine the structural properties of the polymeric matrices (Figure S2, ^1^H NMR spectra of PLLA; Figure S3, ^13^C NMR spectra of PLLA; Figure S4, ^1^H NMR spectra of PLACL; Figure S5, ^13^C NMR spectra of PLACL; Figure S6, ^1^H NMR spectra of PGACL; Figure S7, ^13^C NMR spectra of PGACL, Supplementary Materials). The characteristic signals observed in the spectra were assigned according to the literature, which confirmed the structures of the produced PLLA, PLACL, and PGACL [35,36,37,47].

As seen in Figure S8 (Supplementary Materials), which displays the PLLA carbonyl and methine regions, the only spectral lines that could be assigned represent *iiiii* hexad and *iii* tetrad. Accordingly, our data indicates that transesterification has no effect on the stereoregularity of the produced PLLA. Consequently, the synthesized PLLA exhibited high isotacticity.

**Figure 1:**
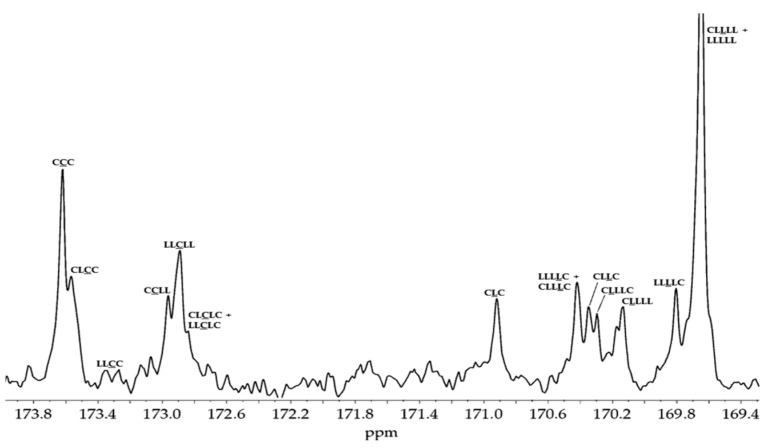
Expansion of the domain of interest in ^13^C NMR spectrum of carbonyl region for PLACL copolymer (M2).

**Figure 2:**
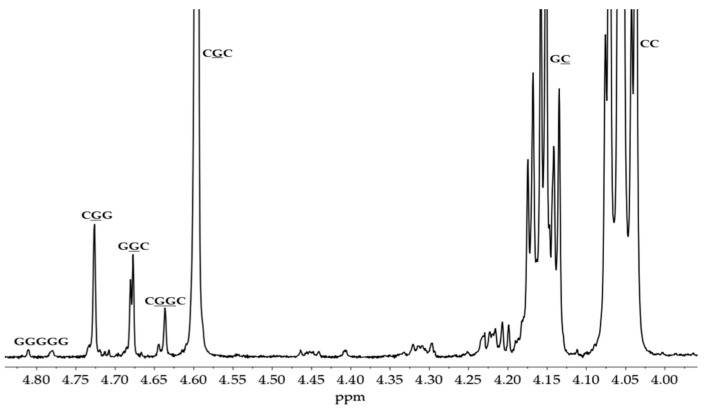
Expansion of the domain of interest in ^1^H NMR spectrum of PGACL copolymer (M3 and M4).

The examination of the copolymer spectra, particularly the ^13^C NMR spectrum of PLACL (M2) (Figure 1) and the ^1^H NMR spectra of PGACL (M3 and M4) (Figure 2), were supported in the identification of spectral lines corresponding to distinct comonomeric sequences within the PGACL and PLACL copolymeric chains. The ^1^H NMR spectra of M3 and M4 revealed the presence of a signal generated from the -*GCapG-* triad, with *G* representing the glycolidyl unit -OCH_2_CO- and *Cap* (*C*) representing the caproyl unit -O(CH_2_)_5_CO-. The presence of this signal indicated that type II intermolecular transesterification occurred during the synthesis. Furthermore, the presence of the -*CapLCap*- triad in the ^13^C NMR spectrum of PLACL, where *L* represents the lactydyl unit -OCH(CH_3_)CO-, suggests that in this case, type II intermolecular transesterification also took place. The distribution of comonomeric units in the polymer chains was calculated according to the literature and using the relevant equations (2–8, Experimental section) [36,37]. The average block lengths, transesterification of the second mode, and degree of randomness are provided in Table 2. The estimated findings indicate that the resultant copolymers have an atactic chain microstructure.

Given that it is commonly known that materials used in the medical and pharmaceutical fields must fulfil certain criteria, the resulting polymer matrices were subjected to biological tests, namely cell viability and hemolysis.

**Figure 3:**
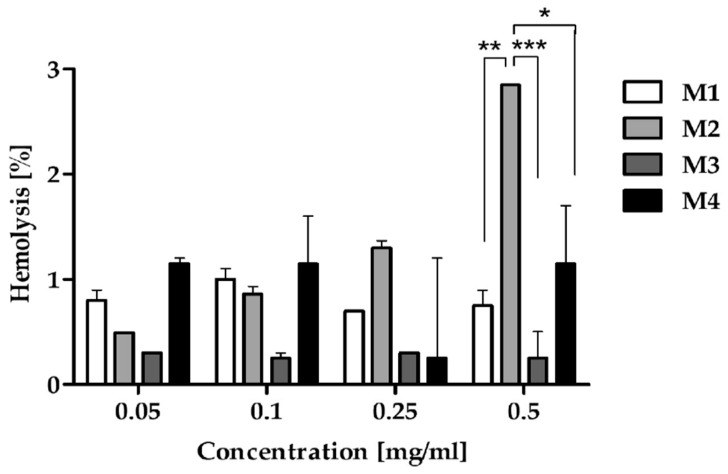
Hemolytic activity of the synthesized biodegradable polymers: M1, M2, M3, and M4 after 1 h of incubation. The graph depicts the level of hemolysis of RBC treated with increasing concentrations of the complex after 1 h of treatment. Two-way ANOVA followed by the Bonferroni post-test was used for statistical analysis. The results were considered statistically significant: * *p* < 0.05; ** *p* < 0.01; *** *p* < 0.005.

The level of RBC hemolysis was matrix-dependent (*p* = 0.0982), but concentration-independent (Figure 3). Among tested polymeric matrices, the lowest hemolysis was induced by M3 (max. 0.3%) followed by M1 (max. 1%) and M4 (max. 1.2%). The highest hemolytic activity was observed for M2 (max. 2.85%). No statistically significant differences in the level of hemolysis were observed between all matrices except the highest concentration. At a concentration of 0.5 mg/mL, M2-induced hemolysis was significantly higher compared to M1, M3, and M4.

A hemolysis assay is an indispensable initial step in evaluating the blood compatibility of polymers to identify severe acute toxic reactions in RBCs in vivo [48]. Furthermore, many studies have reported that in vitro hemolysis assays have good correlations with in vivo toxicity considering the hemolytic effect [49,50]. Importantly, if the hemolysis rate is less than 5%, medicinal products are termed non-hemolysis under national biological safety guidelines. In our study, all tested matrices revealed results of less than 5%, meeting the national regulatory requirements for biological materials and establishing their hemocompatibility for possible intravenous application (Figure 3 and Figure S9, Supplementary Materials).

**Figure 4:**
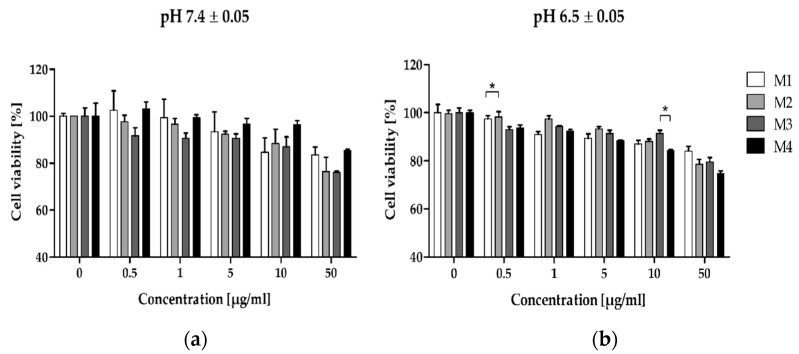
The viability of normal fibroblasts treated for 72 h with the synthesized biodegradable polymers at pH 7.4 ± 0.05 (**a**) and pH 6.5 ± 0.05 (**b**). The graphs depict differences in the susceptibility of cells to the complex. The MTS assay was used to determine the relative cell number. The results are given as mean ± SEM. Two-way ANOVA was used for statistical analysis, followed by Bonferroni post-tests. When the following conditions were met, the results were considered statistically significant: * *p* < 0.05.

As seen in Figure 4, the fibroblasts treated with polymers exhibited comparable concentration-dependent viability. The pH of the cultures had no significant influence on the activity of the polymer samples, except for M4, which caused a greater decrease in viability at pH 6.5 ± 0.05 (see Figure S10, Supplementary Materials). The viability of polymers-treated cells reached approximately 90% viability level of control cells (the concentration ranged from 0.01 to 5 µg/mL). However, at higher concentrations (10–50 µg/mL), the fibroblast viability was reduced to around 80% after 72 h of treatment. A statistically significant difference in cell viability was noted for M1 and M2 matrices at a concentration of 0.5 µg/mL and between M3 and M4 at 10 µg/mL.

### 3.2. Synthesis and Characterization of the Nanosystems Composed of PAMAM Dendrimer/CPT Complex and Biodegradable Polymers

In the second step of the investigation, the synthesized and characterized polymers, as well as the PAMAM dendrimer/CPT complex, were employed to develop nanoparticulate systems for targeted therapy. Based on the previous optimization findings, four nanosystems were produced, each employing a different biodegradable polymer (Table 3).

**Figure 5:**
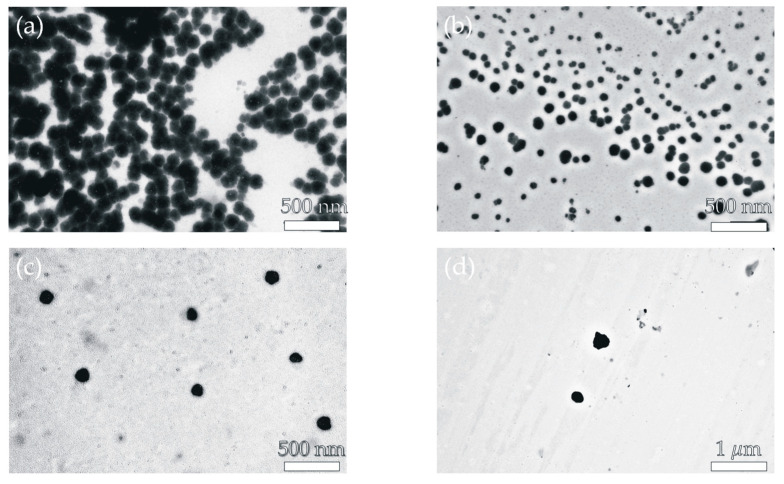
TEM image of: (**a**) NP1 sample, (**b**) NP2 sample, (**c**) NP3 sample, (**d**) NP4 sample.

As shown in Table 3, the *EE* value ranged from 11 ± 2.91 to 27 ± 1.94%. The highest value was observed for the NP2 sample (27 ± 1.94%), and the lowest for the NP4 (11 ± 2.91%). We suspect that these variations are most likely caused by the varied types of polymeric materials used to obtain nanosystems. Furthermore, the DLS analysis indicated that the developed nanosystems had an average size of 190 nm (NP1), 110 nm (NP2), 284 nm (NP3), and 406 nm (NP4). The dispersity values ranged from 0.10 to 0.67, showing a rather broad particle size distribution. Zeta potential experiments indicated that all produced nanoparticulate systems have a positive surface charge (ranging from 5.24 to 24.4 mV), which is most likely explained by the presence of protonated primary amino groups in PAMAM dendrimer macromolecules [51]. It is also important to note that the TEM images of the produced nanomaterials supported the DLS results. Figure 5 shows that the size range of the nanosystems was below 500 nm.

**Figure 6:**
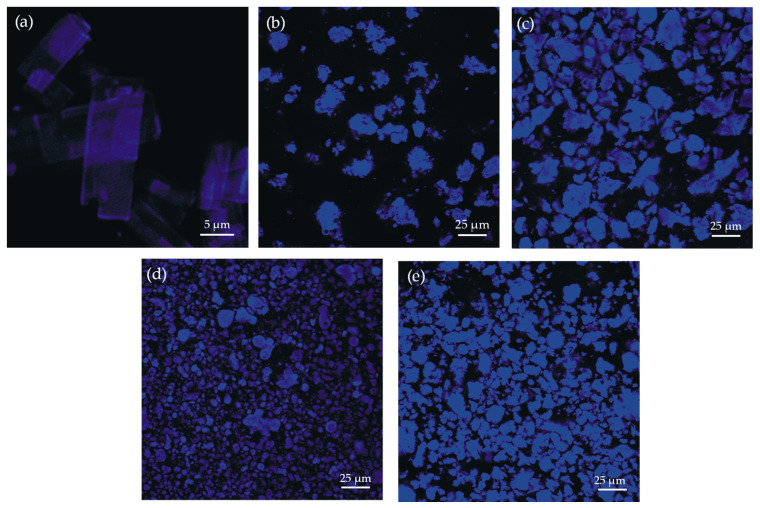
Confocal fluorescence imaging of: (**a**) CPT, (**b**) NP1 sample, (**c**) NP2 sample, (**d**) NP3 sample, (**e**) NP4 sample.

To confirm the presence of CPT more clearly in the prepared nanosystems and roughly determine its location, the confocal microscopy was applied (Figure 6). Given that CPT is a naturally fluorescent substance (Figure 6a) [52], this technique appears to be suitable for this specific application. The confocal microscopic images showed an effective entrapment of this drug into the PAMAM dendrimer cavity as fluorescence was detected in all nanoparticulate materials.

### 3.3. CPT Release Study from the Nanosystems Composed of PAMAM Dendrimer/CPT Complex and Biodegradable Polymmer

**Figure 7:**
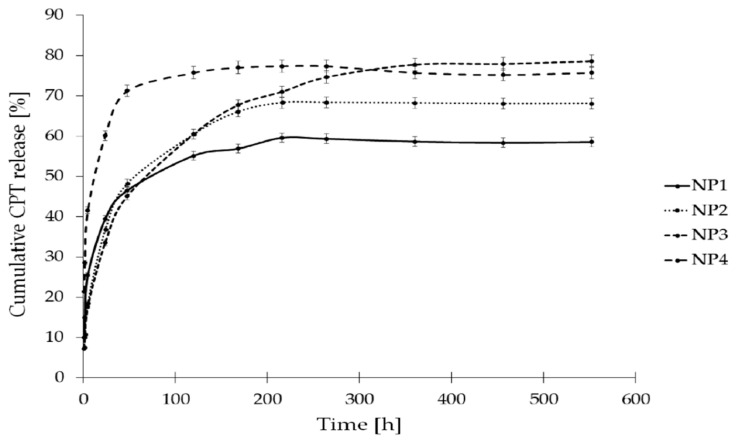
CPT release profiles from the developed nanosystems at pH 6.50 ± 0.05.

**Figure 8:**
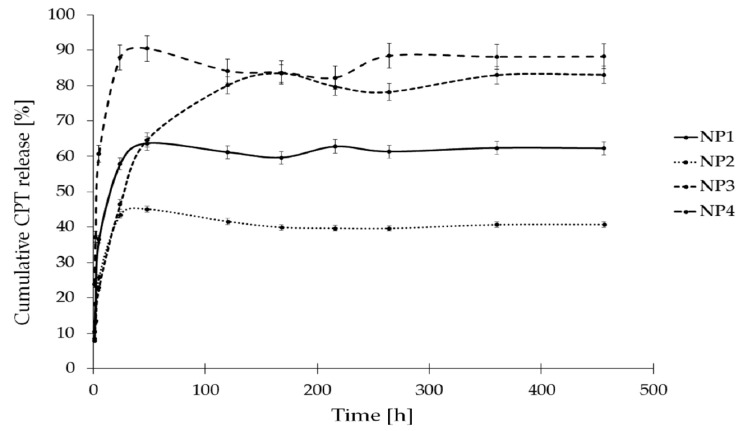
CPT release profiles from the developed nanosystems at pH 7.40 ± 0.05.

The in vitro release study of CPT was conducted for more than 400 h, utilizing phosphate buffer solutions at two distinct pH levels: 7.40 ± 0.05, which simulates physiological conditions found in the bloodstream, and 6.50 ± 0.05, representing the acidic environment characteristic of endosomes in cancer cells. This study aimed to assess stability and elucidate the CPT release characteristics from the developed nanosystems. The drug release profiles for the samples NP1, NP2, NP3, and NP4 are illustrated as a correlation between cumulative drug release and time (Figure 7 and Figure 8). As it can be clearly seen in Figure 7, the drug release rate at a pH of 6.50 ± 0.05 reached a plateau after 120 h for NP4, 216 h for NP1 and NP2, and after 360 h for NP3. The highest drug release rate was observed for the samples NP3 and NP4 (78%), while the NP1 material released only 60% of the active substance. Figure 8 depicts the release rates at a pH of 7.40 ± 0.05. It can be observed that a plateau was achieved after 48 h for the samples NP1, NP2, and NP4, whereas sample NP3 reached its plateau after 168 h. The highest release rate was observed for the NP4 sample (91%), while the NP2 nanosystem demonstrated the lowest drug release rate (45%).

The findings from the two distinct release media are notably consistent, with the highest release rates recorded for the nanosystems obtained using PGACL (NP3 and NP4). This observation may be attributed to the presence of the GL units within the copolymeric chain. The degradation rates of GL copolymers are closely related to the GL composition, as these copolymers exhibit significant hydrophilicity, rendering them more susceptible to hydrolytic degradation [53].

To summarize these findings, it is important to highlight that coating the PAMAM dendrimer/CPT complex with a biodegradable polymer significantly influenced the drug release profile. The time-dependent mechanisms facilitated a gradual and controlled release of the drug from the formulated nanosystems. This phenomenon may be attributed to several factors, including the dissociation during dilution, the competitive displacement of the drug from the PAMAM dendrimer cavity by components of the release medium, the adsorption of the free drug onto the nanoparticle surface, and the diffusion of the free drug from the polymer chain structure [54].

**Figure 9:**
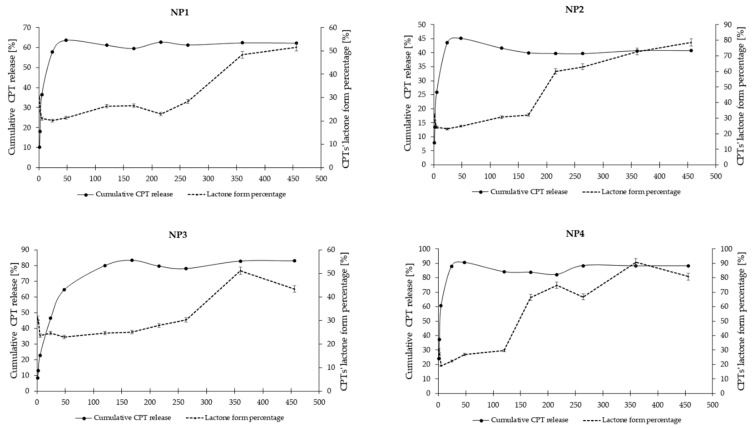
Correlation between cumulative CPT release and percentage of the lactone form of CPT at 7.40 ± 0.05.

**Figure 10:**
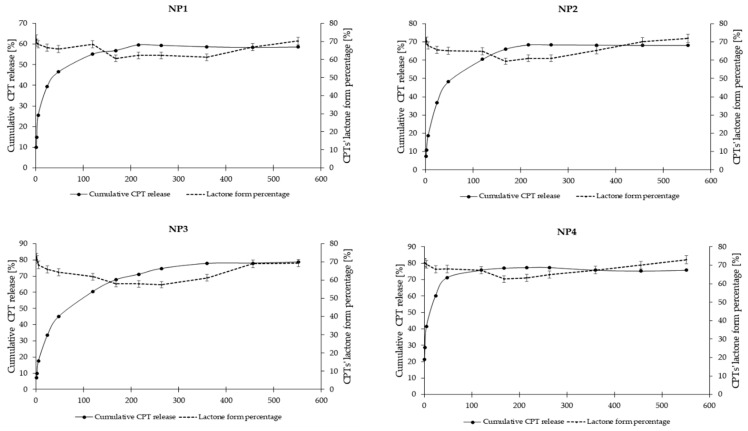
Correlation between cumulative CPT release and percentage of the lactone form of CPT at 6.50 ± 0.05.

The release rates illustrated in Figure 7 and Figure 8 include both the carboxylic form of CPT, which is pharmacologically inactive, and the lactone form, which is pharmacologically active. According to existing literature, these two forms exist in a state of equilibrium [55]. However, the percentage content of the lactone form of CPT is a critical factor to consider, especially in predicting tumor response. Given that physiological conditions facilitate the hydrolysis of the lactone ring, utilizing biodegradable polymers as a vehicle for CPT presents a viable solution to the issue of CPT instability. As can be seen in Figure S11 (Supplementary Materials), both forms of CPT were detected during the release studies conducted at pH 7.4 ± 0.05 (Figure S11a) and pH 6.5 ± 0.05 (Figure S11b). A comparative analysis of the lactone conformation percentage at pH 7.4 ± 0.05 reveals a notable increase in its content over the duration of incubation (Figure 9). This increase is probably attributable to the acidic degradation products of the polymer carriers present in the incubation environment, which effectively lower the pH and enhance the stability of the pharmacologically active form of CPT. A similar trend was observed in the release study conducted at pH 6.5 ± 0.05 (Figure 10). In all samples, the percentage of the lactone form of CPT was significantly higher than what has been reported in the existing literature [55]. Additionally, it is important to note that, in contrast to the findings of Liu and coworkers, who reported no observable conversion from lactone to carboxylate structures in CPT-loaded poly(ω-pentadecalactone-*co*-butylene-*co*-succinate) (PPBS) nanoparticles over a 24 h period [56], our study demonstrates a significant increase in the lactone form of CPT over a duration of 450 h. This increase is particularly pronounced at 6.50 ± 0.05, where all analyzed nanosystems exhibited a lactone form content exceeding 70%.

Based on these findings, it can be concluded that the developed nanoparticulate systems enabled the stabilization of the pharmacologically active form of CPT and provided its protection against hydrolysis, even under physiological conditions.

In the next step, the data collected from the CPT release studies were analyzed using zero-order, first-order, Higuchi, and Korsmeyer–Peppas models to assess the kinetics and mechanism of CPT release from the developed nanosystems (Table 4).

The release kinetics of CPT at a pH of 7.4 ± 0.05 from the nanosystem NP3 conformed to a near-first-order model, as indicated by an R^2^ value of 0.740. Additionally, drug release at a pH of 6.5 ± 0.05 from the samples NP1, NP2, and NP3 exhibited near-first-order kinetics, with R^2^ values of 0.717, 0.779, and 0.877, respectively. It is also likely that CPT was released from the nanosystems NP1, NP2, and NP4 at pH 7.4 ± 0.05, as well as from NP4 at pH 6.5 ± 0.05, following first-order kinetics; however, this conclusion is made with caution due to the relatively low R^2^ values (R^2^ < 0.7). Analysis of the CPT release data using the Higuchi model indicated that CPT was released from the sample NP3 at pH 7.4 ± 0.05 and from all nanosystems at pH 6.5 ± 0.05 via a diffusion mechanism, as evidenced by R^2^ values exceeding 0.7. Furthermore, the analysis of CPT release data from all nanosystems using the Korsmeyer–Peppas model suggested that the transport mechanism was either Fickian (n = 0.23–0.32, R^2^ > 0.7) or non-Fickian (n = 0.45–0.62, R^2^ > 0.9).

### 3.4. Molecular Modeling of the Nanosystems Composed of PAMAM Dendrimer/CPT Complex and Biodegradable Polymer

#### 3.4.1. MODELS Preparation

Molecular simulations of dendrimer-drug complexes, while inherently challenging, provide valuable insights into the conformational flexibility of dendrimers and the atomic-level interactions that occur during the association of dendrimers with drugs. In our prior research, which detailed the molecular modeling of the PAMAM dendrimer/CPT complex [34], we established that the most stable configuration for the PAMAM dendrimer of generation 4.0 corresponds to a molar ratio of 1:3 for PAMAM dendrimer to CPT. This finding aligns with experimental data.

**Figure 11:**
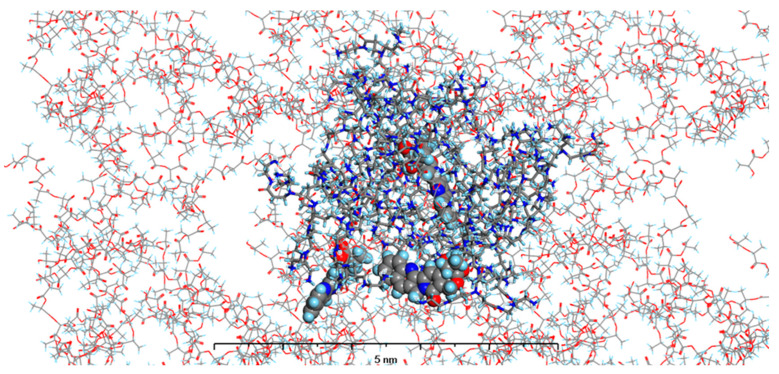
The structure of MODEL 1—PAMAM dendrimer generation 4.0 loaded with three molecules of CPT and surrounded by PLLA matrix. Atom coloring: Hydrogen—light blue, Nitrogen—dark blue, Carbon—grey, Oxygen—red. Rendering: the atoms and bonds of PLLA are rendered as sticks, the atoms of PAMAM dendrimer generation 4.0 are rendered as solid cylinders, CPT atoms are rendered as spheres with radii that are related to the van der Waals radii of its atoms.

**Figure 12:**
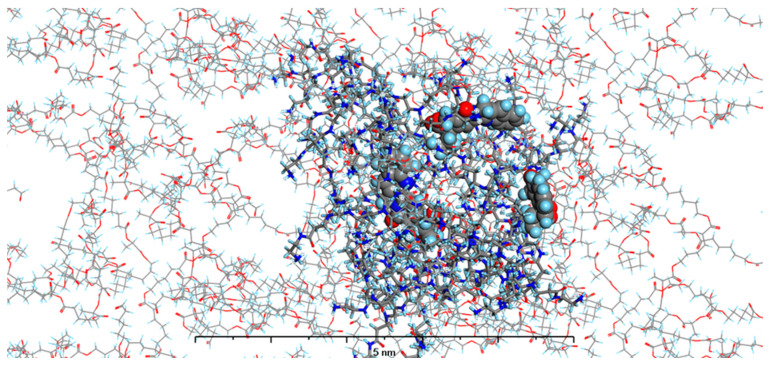
The structure of MODEL 2—PAMAM dendrimer generation 4.0 loaded with three molecules of CPT and surrounded by PLACL matrix. Atom coloring: Hydrogen—light blue, Nitrogen—dark blue, Carbon—grey, Oxygen—red. Rendering: the atoms and bonds of PLLA are rendered as sticks, the atoms of PAMAM dendrimer generation 4.0 are rendered as solid cylinders, CPT atoms are rendered as spheres with radii that are related to the van der Waals radii of its atoms.

**Figure 13:**
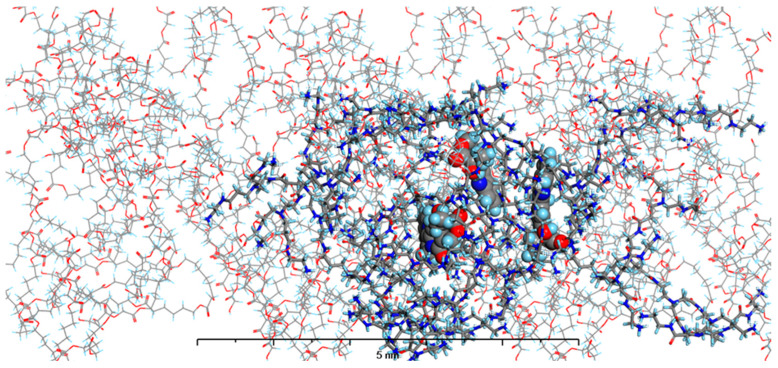
The structure of MODEL 3—PAMAM dendrimer generation 4.0 loaded with three molecules of CPT and surrounded by PGACL 85:15 (*ε*-CL:GL) matrix. Atom coloring: Hydrogen—light blue, Nitrogen—dark blue, Carbon—grey, Oxygen—red. Rendering: the atoms and bonds of PLLA are rendered as sticks, the atoms of PAMAM dendrimer generation 4.0 are rendered as solid cylinders, CPT atoms are rendered as spheres with radii that are related to the van der Waals radii of its atoms.

**Figure 14:**
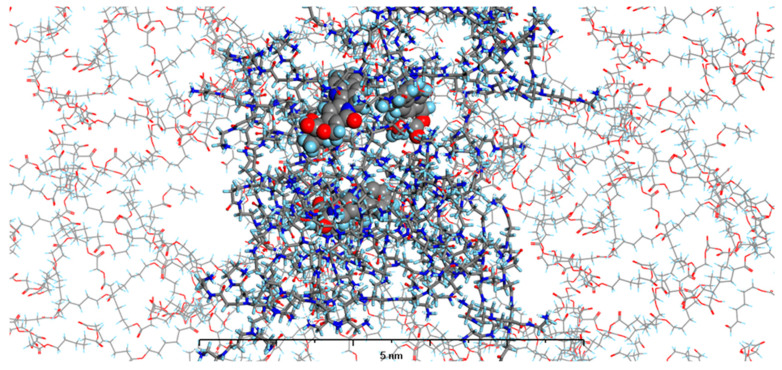
The structure of MODEL 4—PAMAM dendrimer generation 4.0 loaded with three molecules of CPT and surrounded by PGACL 90:10 (*ε*-CL:GL) matrix. Atom coloring: Hydrogen—light blue, Nitrogen—dark blue, Carbon—grey, Oxygen—red. Rendering: the atoms and bonds of PLLA are rendered as sticks, the atoms of PAMAM dendrimer generation 4.0 are rendered as solid cylinders, CPT atoms are rendered as spheres with radii that are related to the van der Waals radii of its atoms.

In our current study, we enhanced our model by incorporating the specific environments for each of the four types of prepared nanosystems. This development led to the creation of MODELS 1–4, which are detailed in the Materials and Methods section. Each of these models consists of a PAMAM dendrimer/CPT complex, featuring three CPT molecules for every PAMAM dendrimer of generation 4.0, all set within a polymer matrix that aligns with the experimental composition. It is important to highlight that, given the size of the experimentally obtained systems, which are several hundred nanometers, we conducted our calculations under periodic boundary conditions. This approach allows for a more accurate representation of the intramolecular forces that influence the structure and behavior of the system. The structures of MODELS 1–4 can be found in Figure 11, Figure 12, Figure 13 and Figure 14.

The structures presented exhibit similarities in the shape and size of the PAMAM dendrimer/CPT complex within the nanosystems, with the exception of MODEL 3. In this particular one, the PAMAM dendrimer structure is notably more branched and less compact, allowing the PGACL chains to easily interweave with the PAMAM dendrimer chains. This suggests that the interactions between the matrix components, PGACL and PAMAM dendrimer, are stronger in this nanosystem, resulting in reduced surface tension and enhanced miscibility. Furthermore, these interactions likely contribute to the aggregation observed in some nanosystems, as confirmed by the DLS graph analysis.

The structures of the MODELS align with the findings from the in vitro CPT release evaluation. MODELS 1 and 2, which feature a more compact arrangement of the PAMAM dendrimer/CPT components, exhibit a notably slower release of CPT in vitro. In contrast, MODELS 3 and 4 have a less dense structure, with copolymer molecules integrated even within the core of the PAMAM dendrimer. This structural difference accounts for the more efficient drug release observed in these two models, particularly at a pH of 7.4 ± 0.05. These insights suggest that the design of the optimized system may play a crucial role in determining drug release characteristics, especially when comparing multiple systems.

#### 3.4.2. Molecular Dynamics (MD) Simulations

Based on the MD simulation results for the MODELS 1–4, chosen geometric features of the complexes were determined, including the mean gyration radius (R_g_), solvent-accessible surface area (SASA), the radial distribution functions G(r), and root-mean-square deviation of atomic positions (RMSD).

Gyration radius (R_g_) represents a measure of how much the polymer chains curl and their average dimensions. Opitz and Wagner used molecular dynamics simulations to study the molecular structure of PAMAM dendrimers and discovered that the gyration radius determined by the experiment was in good agreement with the simulation outcomes [57]. Also, in our previous work, we have successfully determined the R_g_ values using this computational approach [34]. The experimentally determined R_g_ of PAMAM dendrimer generation 4.0 at neutral pH was found to be 22.5 Å [58], while in this study the R_g_ for MODELS 1–4 PAMAM dendrimer/CPT complex were 20.4 Å, 21.0 Å; 24.5 Å, and 23.6 Å, respectively. The decrease of the R_g_ upon complexation, when compared with the free PAMAM dendrimer molecule, was observed in MODELS 1 and 2. This can be explained as resulting from the intermolecular forces (van der Waals, hydrogen bonds) existing between the host and guest (CPT) molecules that are located in the cavity of the PAMAM dendrimer. The expansion of PAMAM dendrimer dimensions observed in MODELS 4, and especially MODEL 3, results from the higher affinity of PAMAM dendrimer molecules towards the polymer matrix particles simulated in that model. Therefore, instead of existing in their spherical shape, the molecules of PAMAM dendrimer tend to increase their specific surface. It also explains the different behavior of nanosystems, observed in the dissolution studies. To confirm this observation, we have calculated the SASA for the nanosystems in each model.

To obtain the SASA, a probe radius of 1.4 Å was used as the van der Waals radius of a water molecule of 2.75 Å. The obtained results were 13,960.65, 14,969.37, 17,326.98 Å^2^, and 16,414.81 Å^2^ for the MODELS 1–4, respectively. These results are comparable to those obtained for PAMAM at pH = 7.0, namely 14,862.4 Å^2^ [59]. Again, similar as for the R_g_, the increase of SASA was observed for MODELS 3 and 4, which results from the more branched shape that the PAMAM dendrimer forms in these systems.

**Figure 15:**
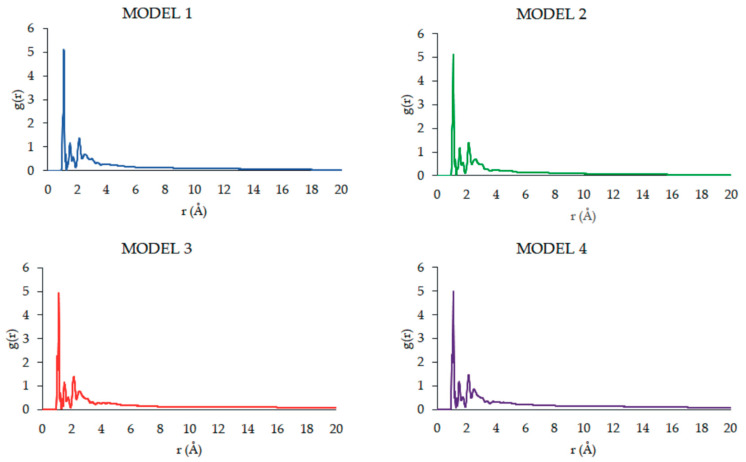
The RDFs between PAMAM and CPT molecules.

The radial distribution function (RDF) represents the features of the microstructure. This function is frequently employed to explore the structure and unique interactions of the condensed matter [60]. Its physical meaning relates to the ratio of the probability density of another atom at a distance from the center atom to the random distribution density. In the g(r)-r diagram (Figure 15), the chemical bonds and hydrogen bond interactions distances are mostly within 3.1 Å, and the non-bonding interactions such as Coulomb forces and van der Waals forces are primarily within 3.1–10.0 Å. The interactions greater than 10.0 Å are very weak. The radial distribution function, g(r), was calculated by averaging the distribution of interatomic vector lengths on a sphere of radius (r) and thickness (Δr). We used Δr = 0.1 Å with the radius, r, of the sphere varying in the range 1–20 Å. The interactions between PAMAM dendrimer and CPT molecules can be analyzed by means of RDFs that were created for each model (Figure 15). The highest peak exists slightly after 1.0 Å, indicating the presence of covalent bonds. Other peaks are visible in the region of 2.0 to 3.5 Å, which means that the H-bonds are formed between PAMAM dendrimer and CPT molecules. As there are not any peaks in the region above 5.0 Å, this indicates no major electrostatic interactions. Also, the RDFs were found to be similar, regardless of the studied model.

**Figure 16:**
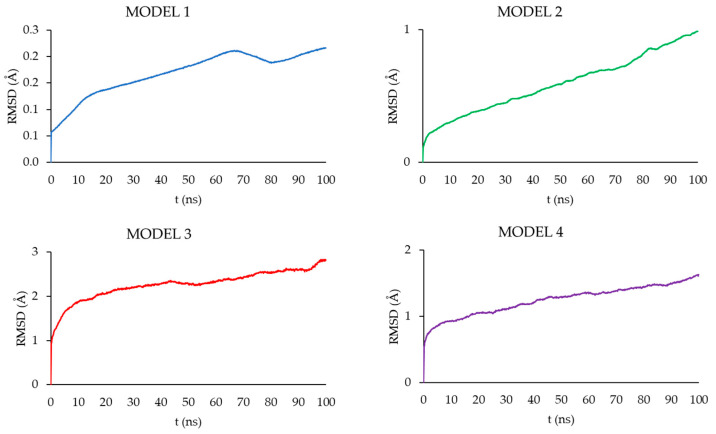
RMSD plots obtained for CPT in MODELS 1–4 during 100 ns MD simulation.

Figure 16 shows the root-mean-square deviation of atomic positions (RMSD) plots obtained for CPT in the studied models during the 100 ns MD simulations. RMSD analysis can indicate the dynamic stability of a molecule in a studied system; larger values of RMDS indicate more intense molecular motions and more efficient diffusion. All the models studied showed an increase in RMSD throughout the simulation time. However, the values reached after 100 ns depend on the model studied. The highest final RMSD was observed in MODEL 3, and the lowest in MODEL 1, 2.5 Å and 0.2 Å, respectively. This indicates that the polymer matrix has a major influence on the behavior of CPT in the nanosystem, which was also observed experimentally.

## 4. Conclusions

The development of DDSs has been influential in transforming innovative therapies into effective medical interventions. As the therapeutic environment has progressed, these systems and their underlying technologies have quickly evolved to accommodate the changing requirements of drug delivery. The physicochemical attributes of small molecules largely determine their delivery mechanisms, which in turn influence their bioavailability. Therefore, drug delivery strategies prioritize not only the improvement of solubility, but also the management of release profiles, the augmentation of therapeutic effects, and the customization of pharmacokinetic behaviors.

In line with this focus, our research concentrated on the development of forward-looking PAMAM dendrimer/biodegradable polymer nanosystems intended for the delivery of CPT. Despite the fact that this drug has been recognized as one of the most promising anticancer agents in recent years, challenges related to its physiological stability and solubility remain unresolved. Initially, four biodegradable homo- and copolymeric matrices were synthesized and extensively characterized, focusing on the microstructural aspects of their chains. The biological assessment conducted on these biodegradable materials, specifically hemolysis tests, revealed that all matrices exhibited hemolysis rates below 5%, thereby conforming to national safety standards for biological materials. Furthermore, fibroblasts exposed to the synthesized polymeric matrices demonstrated viability that was comparable and dependent on concentration. In the subsequent phase of our research, four nanosystems with diameters ranging from 110 to 406 nm and an entrapment efficiency of up to 27% were developed. These nanoparticulate systems were formulated from a PAMAM dendrimer/CPT complex, which was subsequently coated with an appropriate biodegradable matrix. Notably, confocal microscopy confirmed the successful entrapment of CPT within the PAMAM dendrimer cavity, as evidenced by the detection of fluorescence from the drug in all nanoparticulate systems. The research demonstrated that CPT was released from the engineered nanosystems in a sustained and controlled manner, exhibiting near-first-order and first-order kinetics without any burst release effect. Additionally, the biodegradable polymer matrices played a significant role in shaping the drug release profile. Notably, the nanoparticulate systems we have developed facilitated the stabilization of the pharmacologically active form of CPT, offering protection against hydrolysis even under physiological conditions. Through molecular modeling techniques, we constructed four MODELS, each comprising a PAMAM dendrimer/CPT complex with three CPT molecules associated with each generation 4.0 PAMAM dendrimer, all integrated within a polymer matrix that corresponds to the experimental setup. Our analysis revealed that the structures of the MODELS displayed similarities in the shape and size of the PAMAM dendrimer/CPT complex within the nanosystems, with MODEL 3 being an exception. In this particular model, the PAMAM dendrimer structure was significantly more branched and less compact, which facilitates the interweaving of PGACL matrix chains with the PAMAM dendrimer chains. This indicates that the interactions between the matrix components, PGACL and PAMAM dendrimer, were more robust in this nanosystem, leading to decreased surface tension and improved miscibility. The structural characteristics of the MODELS were consistent with the results obtained from the in vitro evaluation of CPT release.

In summary, we can assert that the nanoparticulate systems developed may serve as a novel platform for the delivery of significant hydrophobic anticancer drugs. Consequently, these nanomaterials hold great promise as delivery systems for cancer therapy, and further investigation of the in vivo behavior of these nanoparticulate materials is warranted in animal models.

## Figures and Tables

**Table 1:** Homo- and copolymerization of CL, GL, and LA. The characteristics of the obtained biodegradable polymers.

Code	Matrix	Monomer Molar Ratio	Yield [%]	*Conv*_i_ ^a^ [%]	*M_n_* ^b^ [g/mol]	*Ɖ* ^b^
M1	PLLA poly(L-lactide)	LA = 1.0	78	97	13,600	1.23
M2	PLACL poly(L-lactide-*co*-ε-caprolactone)	LA = 0.40 CL = 0.60	78	83 (LA) 82 (CL)	8700	1.77
M3	PGACL poly(glycolide-*co*-ε-caprolactone)	CL = 0.85 GL = 0.15	83	86 (CL) 74 (GL)	16,100	1.57
M4		CL = 0.90 GL = 0.10	90	85 (CL) 63 (GL)	21,300	1.52

^a^—calculated from ^1^H NMR; ^b^—determined by SEC-MALLS.

**Table 2:** Analysis of the chain microstructure of the synthesized copolymers.

Code	Polymer	Average Block Length	$T_{II}$	*R*
M2	poly(L-lactide-*co*-ε-caprolactone) PLACL/40:60	$L_{LL}^{e}$ = 1.27 $L_{Cap}^{e}$ = 0.99	0.49	1.00
M3	poly(glycolide-*co*-ε-caprolactone) PGACL/15:85	$L_{GG}^{e}$ = 1.67 $L_{Cap}^{e}$ = 4.43	0.73	0.60
M4	poly(glycolide-*co*-ε-caprolactone) PGACL/10:90	$L_{GG}^{e}$ = 1.46 $L_{Cap}^{e}$ = 8.81	0.94	0.68

$L_{LL}^{e}$—experimental average length of lactydyl blocks; $L_{Cap}^{e}$—experimental average length of caproyl blocks; $L_{GG}^{e}$—experimental average length of glycolidyl blocks; $T_{II}$—yield of the second mode of transesterification; *R*—the degree of randomness.

**Table 3:** Characterization of the developed nanosystems composed of PAMAM dendrimer/CPT complex and biodegradable polymer.

Code	Polymer Used	Entrapment Efficiency (*EE*) ^a^ [%]	Drug Loading (*DL*) ^a^ [%]	Size ^b^ [nm]	Zeta Potential ^b^ [mV]	PDI ^b^
NP1	PLLA (M1)	25 ± 2.51	16 ± 1.35	190	5.24 ± 2.34	0.51
NP2	PLACL/40:60 (M2)	27 ± 1.94	17 ± 0.94	110	34.40 ± 2.14	0.52
NP3	PGACL/15:85 (M3)	22 ± 2.42	14 ± 1.78	284	23.43 ± 3.11	0.67
NP4	PGACL/10:90 (M4)	11 ± 2.91	7 ± 3.59	406	26.83 ± 1.79	0.10

^a^—determined by HPLC; ^b^—determined by DLS.

**Table 4:** Analysis data of CPT release from obtained NPs.

Sample	pH	Zero-Order Model	First-Order Model	Higuchi Model	Kosmeyer-Peppas Model		Drug Transport Mechanism
		*R* ^2^	*R* ^2^	*R* ^2^	*R^2^*	*n*	
NP1 NP2 NP3 NP4	7.4 ± 0.05 7.4 ± 0.05 7.4 ± 0.05 7.4 ± 0.05	0.465 0.400 0.620 0.401	0.542 0.445 0.740 0.575	0.688 0.593 0.845 0.616	0.915 0.707 0.993 0.999	0.45 0.23 0.53 0.62	non-Fickian transport Fickian diffusion non-Fickian transport non-Fickian transport
NP1 NP2 NP3 NP4	6.5 ± 0.05 6.5 ± 0.05 6.5 ± 0.05 6.5 ± 0.05	0.628 0.684 0.758 0.517	0.717 0.779 0.877 0.653	0.844 0.893 0.939 0.744	0.952 0.987 0.992 0.978	0.32 0.45 0.45 0.32	Fickian diffusion non-Fickian transport non-Fickian transport Fickian diffusion

## Data Availability

The data presented in this study are available on request from the corresponding author.

## Supplementary Materials

The following supporting information can be downloaded at: https://www.mdpi.com/article/10.3390/pharmaceutics16111482/s1, Figure S1: ^1^H NMR spectrum of the synthesized PAMAM dendrimer/CPT complex, Figure S2: ^1^H NMR spectrum of the PLLA (matrix M1), Figure S3: ^13^C NMR spectrum of the PLLA (matrix M1), Figure S4: ^1^H NMR spectrum of the PLACL 40:60 (matrix M2), Figure S5: ^13^C NMR spectrum of the PLACL 40:60 (matrix M2), Figure S6: ^1^H NMR spectrum of the PGACL (matrices M3 and M4), Figure S7: ^13^C NMR spectrum of the PGACL (matrices M3 and M4), Figure S8: Expansion of the domain of interest in ^13^C NMR spectrum of PLLA (matrix M1), Figure S9: Hemolytic activity of individual matrices. The graph depicts the level of hemolysis of RBC treated with increasing concentrations of the complex after 1 h of treatment. Statistical analysis using two-way ANOVA followed by the Bonferroni post-test revealed no significant differences (*p* > 0.05) between the obtained results, Figure S10: The viability of normal fibroblasts treated for 72 h with individual matrices at pH 7.4 ± 0.05 and 6.5 ± 0.05. The graphs depict differences in the susceptibility of cells to the complex. The MTS assay was used to determine the relative cell number. The results are given as mean ± SEM. Two-way ANOVA was used for statistical analysis, followed by Bonferroni post-tests. When the following conditions were met, the results were considered statistically significant: * *p* < 0.05; ** *p* < 0.01; *** *p* < 0.005, Figure S11: HPLC chromatograms of the CPT released in the lactone and carboxylic forms (a) pH = 7.4 ± 0.05; (b) pH = 6.5 ± 0.05.
