# Supplementary material for: Future-Oriented Nanosystems Composed of Polyamidoamine Dendrimer and Biodegradable Polymers as an Anticancer Drug Carrier for Potential Targeted Treatment
Source: Pharmaceutics. 2024 Nov 20;16(11):1482. doi: 10.3390/pharmaceutics16111482 (PMC11597463; doi:10.3390/pharmaceutics16111482)
Supplement: Supplementary file 1 [file pharmaceutics-16-01482-s001.zip › pharmaceutics-3306827-supplementary.pdf]

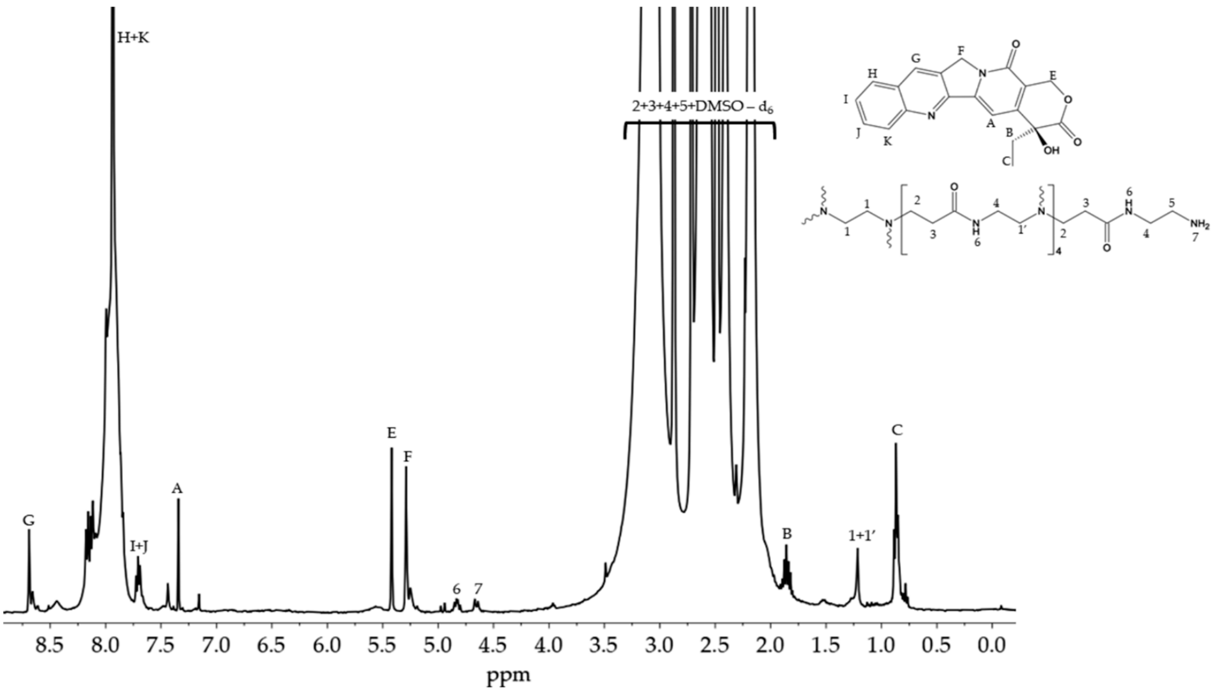

Figure S1. ^1H NMR spectrum of the synthesized PAMAM dendrimer/CPT complex.

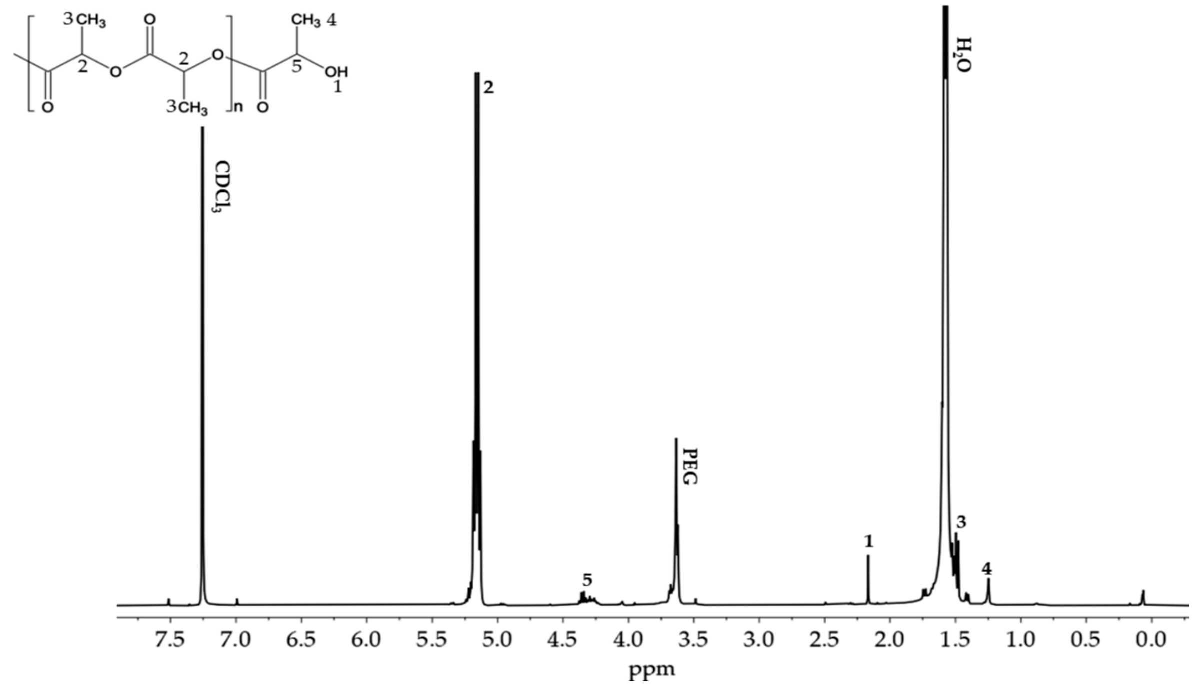

Figure S2. ^1H NMR spectrum of the PLLA (M1).

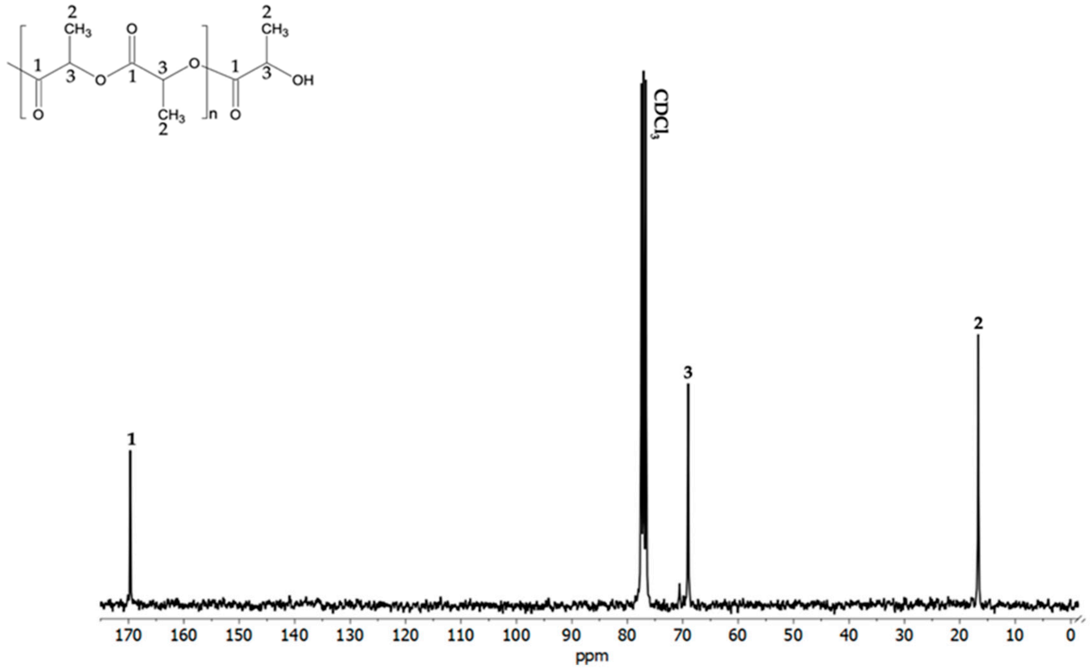

Figure S3. ^{13}C NMR spectrum of the PLLA (M1).

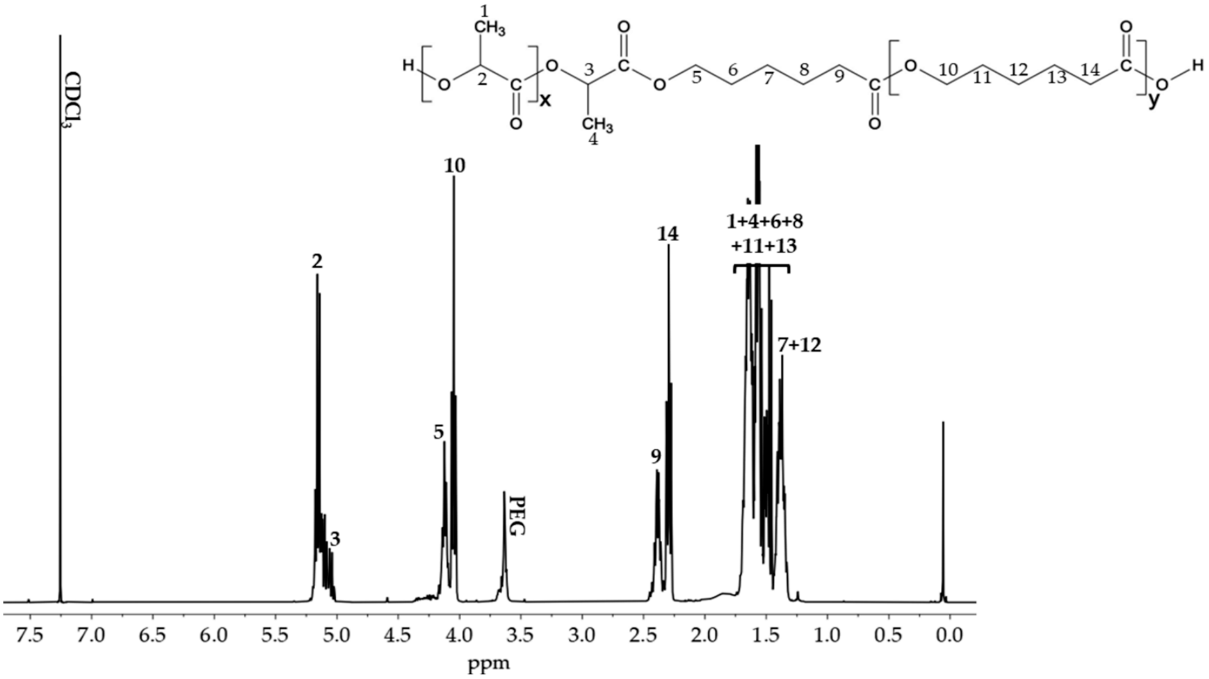

Figure S4. ^{1}H NMR spectrum of the PLACL 40:60 (M2).

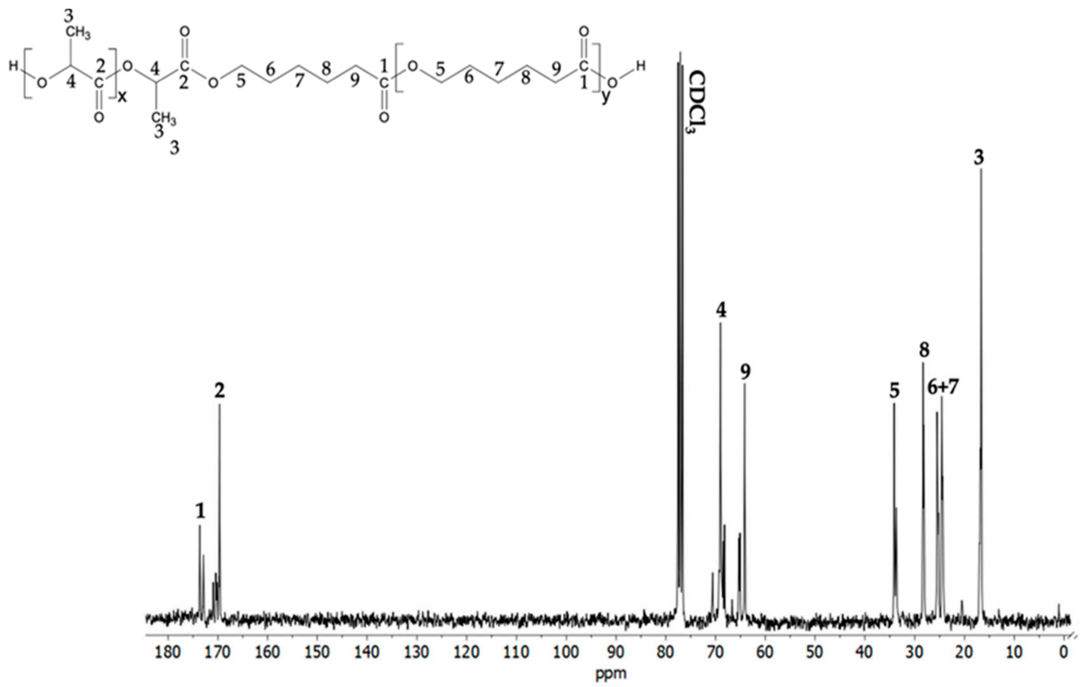

Figure S5. ^{13}C NMR spectrum of the PLACL 40:60 (M2).

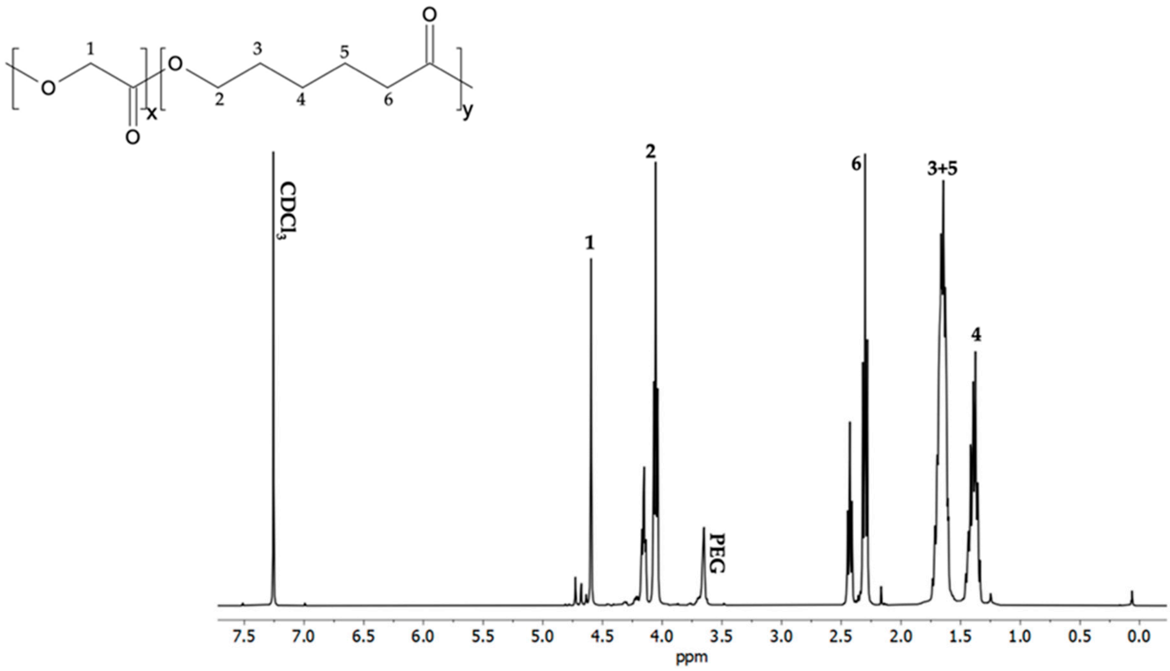

Figure S6. ^{1}H NMR spectrum of the PGACL (M3 and M4).

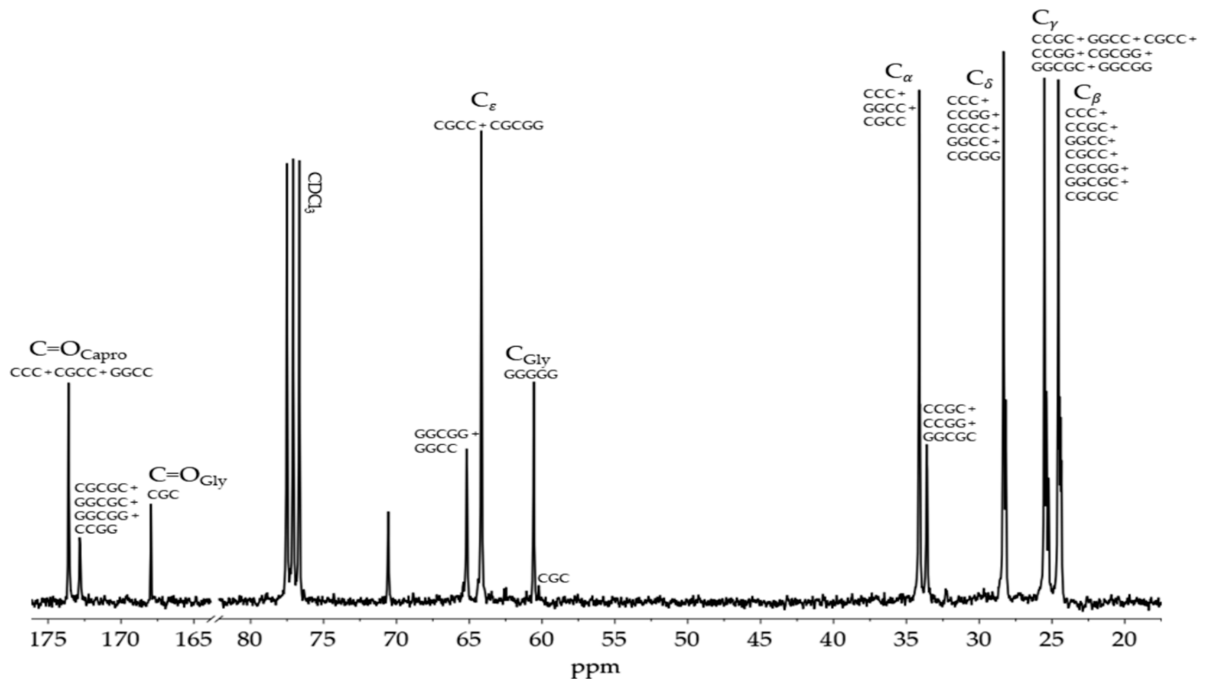

Figure S7. ^{13}C NMR spectrum of the PGACL (M3 and M4).

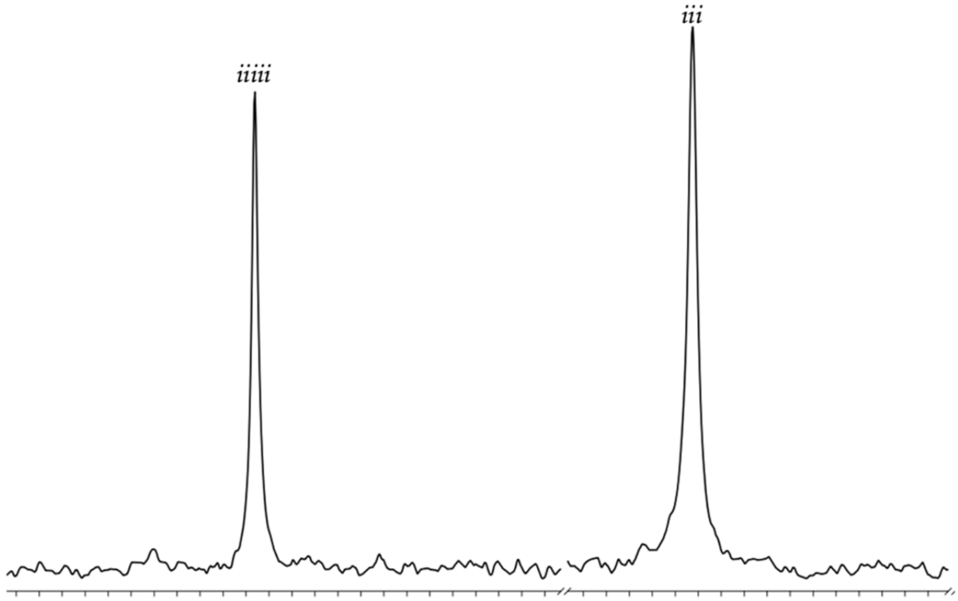

Figure S8. Expansion of the domain of interest in ^{13}C NMR spectrum of PLLA (M1).

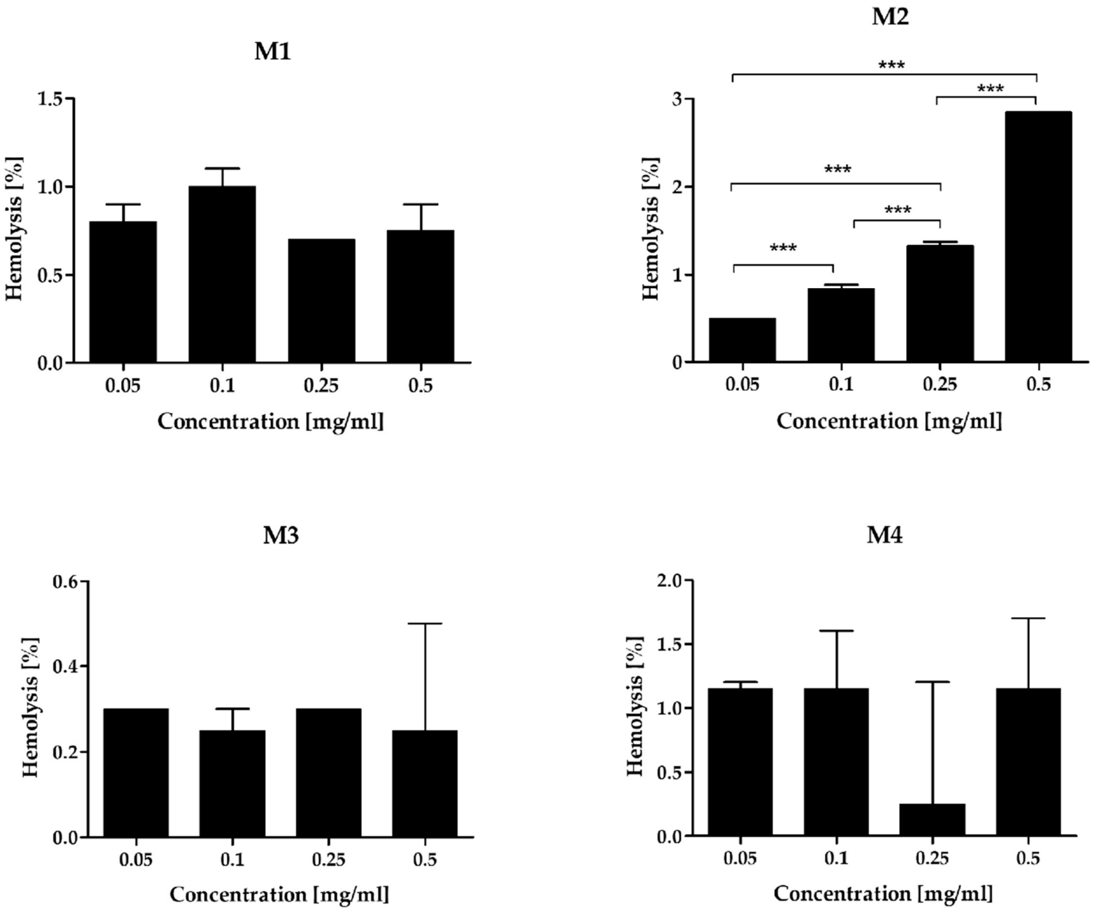

Figure S9. Hemolytic activity of individual polymeric samples. The graph depicts the level of hemolysis of RBC treated with increasing concentrations of the complex after 1 h of treatment. Statistical analysis using two-way ANOVA followed by the Bonferroni post-test revealed no significant differences ($P > 0.05$) between the obtained results.

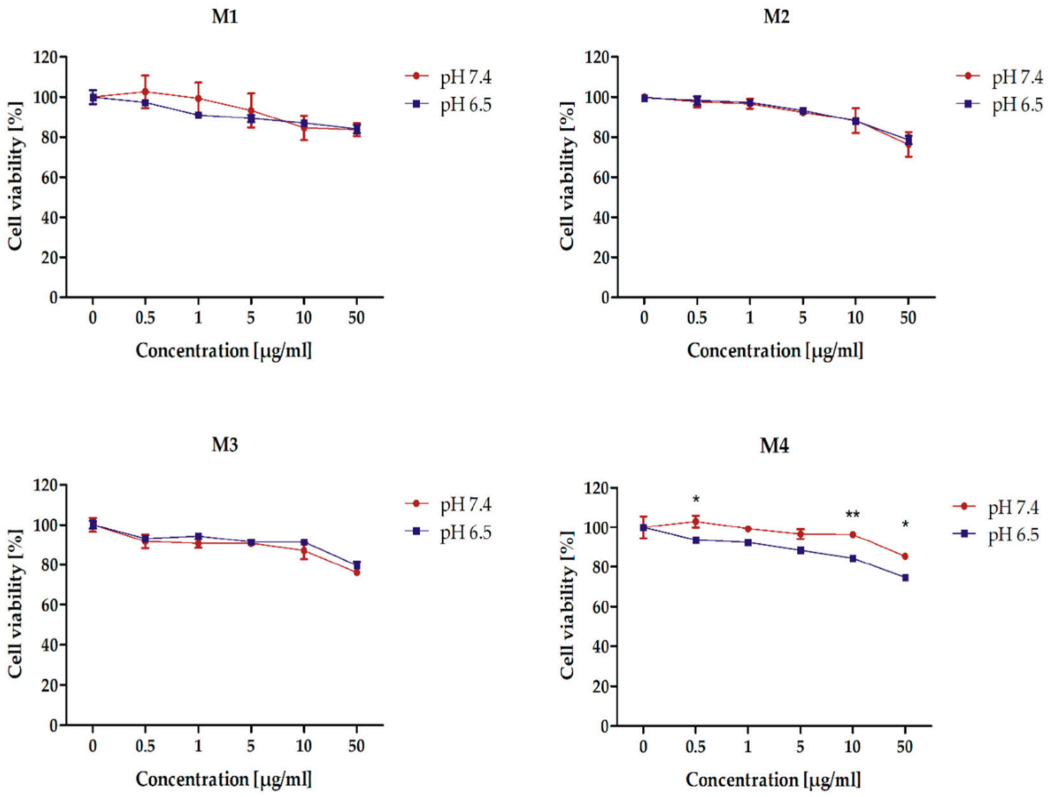

Figure S10. The viability of normal fibroblasts treated for 72 h with individual polymeric samples at pH 7.4±0.05 and 6.5±0.05. The graphs depict differences in the susceptibility of cells to the complex. The MTS assay was used to determine the relative cell number. The results are given as mean ± SEM. Two-way ANOVA was used for statistical analysis, followed by Bonferroni post-tests. When the following conditions were met, the results were considered statistically significant: *$P<0.05$; **$P<0.01$; ***$P<0.005$.

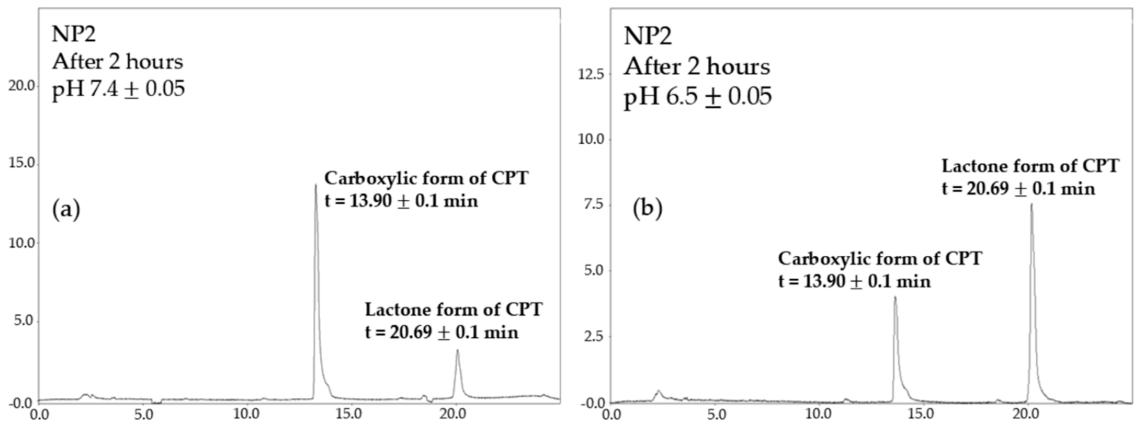

Figure S11. HPLC chromatograms of the CPT released in the lactone and carboxylic forms (a) pH = 7.4 ± 0.05; (b) pH = 6.5 ± 0.05.
